# Transient Hypothyroidism During Lactation Alters the Development of the Corpus Callosum in Rats. An *in vivo* Magnetic Resonance Image and Electron Microscopy Study

**DOI:** 10.3389/fnana.2020.00033

**Published:** 2020-06-26

**Authors:** Federico Salas-Lucia, Jesús Pacheco-Torres, Susana González-Granero, José Manuel García-Verdugo, Pere Berbel

**Affiliations:** ^1^Departamento de Histología y Anatomía, Facultad de Medicina, Universidad Miguel Hernández (UMH), Sant Joan d’Alacant, Spain; ^2^Instituto de Neurociencias de Alicante, UMH – Consejo Superior de Investigaciones Científicas, Sant Joan d’Alacant, Spain; ^3^Laboratorio de Neurobiología Comparada, Instituto Cavanilles de Biodiversidad y Biología Evolutiva, Universitat de València - Centro de Investigación Biomédica en Red sobre Enfermedades Neurodegenerativas (CIBERNED), Valencia, Spain

**Keywords:** neocortical development, thyroid hormones, iodine diet, congenital hypothyroidism, psychiatric diseases, autism, attention deficit/hyperactivity disorder

## Abstract

Magnetic resonance imaging (MRI) data of children with late diagnosed congenital hypothyroidism and cognitive alterations such as abnormal verbal memory processing suggest altered telencephalic commissural connections. The corpus callosum (CC) is the major inter-hemispheric commissure that contra-laterally connects neocortical areas. However, in late diagnosed neonates with congenital hypothyroidism, the possible effect of early transient and chronic postnatal hypothyroidism still remains unknown. We have studied the development of the anterior, middle and posterior CC, using *in vivo* MRI and electron microscopy in hypothyroid and control male rats. Four groups of methimazole (MMI) treated rats were studied. One group, as a model for early transient hypothyroidism, was MMI-treated from postnatal day (P) 0 to P21; some of these rats were also treated with L-thyroxine (T4) from P15 to 21. Another group modeling chronic hypothyroid, were treated with MMI from P0 to 150 and from embryonic day 10 to P170. The results obtained from these groups were compared with same age control rats. The normalized T2 signal obtained using MRI was higher in MMI-treated rats and correlated with a low number and percentage of myelinated axons. The number and density of myelinated axons decreased in transient and chronic hypothyroid rats at P150. The g-ratio (inner to outer diameter ratio) and the estimated conduction velocity of myelinated axons were similar between MMI-treated and controls, but the conduction delay decreased in the posterior CC of MMI-treated rats compared to controls. These data show that early postnatal transient and chronic hypothyroidism alters CC maturation in a way that may affect the callosal transfer of information. These alterations cannot be reversed after delayed T4-treatment. Our data support the findings of neurocognitive delay in late T4-treated children with congenital hypothyroidism.

## Introduction

In humans, congenital hypothyroidism is commonly caused by agenesis, dysgenesis (including ectopia) and dysfunction of the thyroid gland, causing neurological and psychiatric deficits, such as intellectual disability, spasticity, and disturbances in gait and co-ordination (Dussault and Ruel, 1987; Rovet et al., 1992; Rovet, 1999, 2002; Brown, 2012; Clairman et al., 2015; Krude et al., 2015; Léger, 2015; Aycan et al., 2017).

Screening programs are crucial for the early detection and treatment of congenital hypothyroidism in an attempt to avoid the severe neurological and mental diseases that results from late diagnosis, although neurological deficits may persist even when congenital hypothyroidism is diagnosed at birth (Rovet et al., 1992; Rovet, 2005). Altered language, motor, auditory, and visual functions in children with early postnatal thyroid hormone insufficiency are associated with changes in the thickness of neocortical areas (Clairman et al., 2015). Despite a generalized implementation of screening programs in developed countries, the incidence of primary transient congenital hypothyroidism is on the increase in some countries, particularly cases with mild dysfunction of the thyroid gland (Pearce et al., 2010; Léger et al., 2015). The causes for this remain under discussion but may be related to delays in the onset of L-thyroxine (T4) treatment (Rovet et al., 1992), screening thresholds (Olivieri et al., 2013; Léger et al., 2014) and to iodine deficiency during lactation and early infancy (Morreale de Escobar and Escobar del Rey, 1980, 2003; Sava et al., 1984; Köhler et al., 1996; Pearce et al., 2004; Berbel et al., 2007; Walker et al., 2007; Berbel and Morreale de Escobar, 2011).

The Krakow declaration on iodine has reported that Europe is an iodine deficient continent, where up to 50% of newly born children in Europe are exposed to an iodine deficiency which could compromise their thyroid function and brain development (EUthyroid Consortium, 2018). In southern Italy, a mildly iodine-deficient region, hypothyroidism associated with postpartum thyroiditis can affect up to 3.9% of women (Stagnaro-Green et al., 2011b), and more importantly, postpartum thyroiditis is usually only diagnosed by the sixth month postpartum (Lazarus, 2011; Stagnaro-Green et al., 2011b), effectively at the end of the nursing period recommended by the WHO and UNICEF (Stagnaro-Green et al., 2011a).

In neonates, thyroid hormones are of importance in the control of evolutive and developmental events leading to the consolidation of functional cortical circuits, including the myelination of callosal axons (Berbel et al., 2014). The corpus callosum (CC) connects the vast majority of neocortical areas and is fundamental for the reciprocal transfer of information between the cerebral hemispheres and their normal function (Innocenti and Price, 2005). Developmental alterations of telencephalic commissures have been described in relation with neuro-psychiatric diseases such as dyslexia (Hynd et al., 1995), attention deficit hyperactivity disorder (ADHD; Hynd et al., 1991; Humphreys et al., 2016), autism spectrum disorder (ASD; Piven et al., 1997), and schizophrenia (Innocenti et al., 2003; Guo et al., 2013; Kikinis et al., 2015). Some of these diseases have been found in children with congenital hypothyroidism receiving low doses of T4 treatment (Rovet, 2014). Any factor altering the normal development of telencephalic commissures must be considered as potentially increasing the risk of children developing neurologic and psychiatric diseases.

In the majority of mammals (including humans), the refinement (i.e., loss of transitory connections) and maturation (i.e., myelination) of the callosal connections, as observed in cats and monkeys, mostly occurs at an early postnatal age (Berbel and Innocenti, 1988; Gravel et al., 1990; LaMantia and Rakic, 1990; Berbel et al., 1994). In control rats, myelination of callosal axons begins by P12 and the number of myelinated axons increases, reaching a plateau by P150 in the anterior and middle CC, with no similar plateau being reached in posterior CC by P184 (Berbel et al., 1994). Chronic hypothyroidism at the onset of corticogenesis causes alterations in the distribution of callosal projection neurons and a 76% reduction in the myelinated axon number of the CC (Gravel et al., 1990; Berbel et al., 1994), while data from transient and chronic postnatal hypothyroid rats are unknown.

Magnetic resonance imaging (MRI) studying changes in the gross anatomy of CC genu (anterior region of the CC) in children born to women who were hypothyroid at different periods during their pregnancy, has revealed the importance of maternal thyroid hormones for the normal development of interhemispheric connections (Samadi et al., 2015). MRI and electron microscopy (EM) in rats has shown decreased myelination and increased T_2_-ratio (T_2_r) in the anterior commissure of transient and chronic postnatal hypothyroid rats (Lucia et al., 2018). T_2_r is a value (see section “Materials and Methods”) that decreases with the myelination of axons and is used as a velocity conduction index for myelinated axons. These data suggest that early postnatal hypothyroidism, as occurs in congenital hypothyroidism due to iodine deficiency, can arrest myelination and increase T_2_r values in the CC.

Our aim was to study the postnatal development of the CC in transient and chronic hypothyroid rats using EM and *in vivo* MRI. During the first half of the lactating period, transient postnatal hypothyroid rats mimic a delayed diagnosis of congenital hypothyroidism, whilst postnatal rats chronically hypothyroid from birth mimic a postnatal iodine deficiency. We have studied callosal zones containing axons from motor (anterior), somatosensory (middle), auditory, and visual (posterior) cortical areas. We have explored a possible relationship between MRI and quantitative EM data (unmyelinated and myelinated axon number and diameter) using regression functions. The g-ratio (inner to outer diameter ratio) and conduction velocity of myelinated axons were also estimated. The degree of CC damage and recovery following delayed T4 treatment in transient hypothyroid and postnatal hypothyroid rats has been compared to chronic gestational hypothyroid and control rats. Control rats have been used to find non-linear regression functions between quantitative EM and RMI data, which were then verified using chronic gestational hypothyroid rats. Our study is particularly relevant to the clinical case of children late diagnosed with congenital hypothyroidism or those growing up in an iodine deficient environment and showing an impaired neurocognitive development which implicates callosal connections.

## Materials and Methods

### Ethics Statement

Animal care and drug administration were performed under veterinary control according to European Union Directive 86/609/EEC and with approval from the Ethics Committee of the University Miguel Hernández of Elche and the Genèralitat Valènciana, València, Spain.

### Animals and Treatments

The experimental rats used in this study are the same as those used in a previous work on anterior commissure development (Lucia et al., 2018), thereby allowing us to optimize the number of rats sacrificed. Here we present an abbreviated methodology, with a more detailed explanation when necessary, and include differences in the areas studied and sample size. Material was stored at 4°C in 0.05% sodium azide in 0.1M phosphate buffer (PB; pH 7.3–7.4).

As in our previous study, rats were housed in animal quarters with controlled temperature (22–24°C) and light/darkness cycle (14/10 h, respectively). Young females (250–300 g weight) were mated at the embryonic day (E) 0. Hypothyroidism was induced by adding 0.02% methimazole (MMI, Sigma-Aldrich Co., St. Louis, MO, United States) to drinking water. Only male pups were used in this study since gender-based structural differences in the CC have been previously reported. For instance, the percentage of myelinated axons is lower in the genu (Juraska and Kopcik, 1988; Kim and Juraska, 1997) and splenium (Mack et al., 1995) in female rats, and the callosal axon diameter and g-ratio values are higher in male rats (Pesaresi et al., 2015).

**FIGURE 1:**
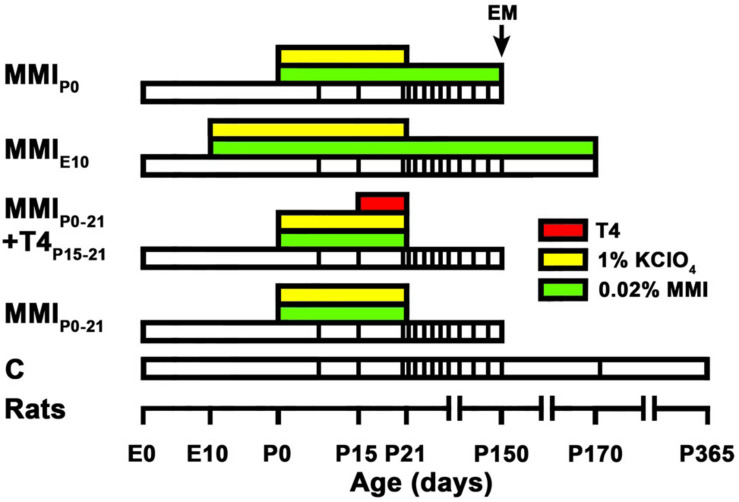
Experimental groups and treatments. The bar chart shows the different treatments of the experimental groups studied. Chronic (MMI_*E*__10_ and MMI_*P*__0_) and transient (MMI_*P*__0__–__21_ and MMI_*P*__0__–__21_ + T4_*P*__15__–__21_) hypothyroid, and control (C) rats were treated (see color key) during their lifespan (white bars), time scale shown on the horizontal axis. The vertical lines within each lifespan indicate the age at which *in vivo* MRI scans were taken. All MMI pups were also treated with 1% KClO_4_ up to P21, to additionally block thyroid function during fetal and lactating periods. Four pups (2 pups per litter per experimental group) were sacrificed at P150 for EM study. The last MRI scan was taken immediately prior to sacrifice at P150 in all groups except for C and MMI_*E*__10_ rats that were not processed for EM but scanned additionally at P170 (C and MMI rats) and P365 (C rats). Data and modified figure legend from Lucia et al. (2018).

Four groups of MMI treated rats were studied, as well as a control group (Figure 1). The MMI_*E*__10_ group was treated from E10 to postnatal day (P) 170, and then sacrificed (at 150, four rats were sacrificed for EM; see below). MMI_*P*__0__–__21_ and MMI_*P*__0_ groups were treated from P0 to P21 and from P0 to P150, respectively, both being sacrificed at P150. The MMI_*P*__0__–__21_ + T4_*P*__15__–__21_ group had the same treatment as MMI_*P*__0__–__21_ pups with the addition of L-thyroxine (T4) (2.4 μg/100 g of body weight; Escobar-Morreale et al., 1995) per day from P15 to P21 (sacrificed at P150). T4 was administered subcutaneously using osmotic mini-pumps (ALZET^®^, model 2001; Alza Corporation, Mountain View, CA), which have a continuous pumping rate of 1 μl/h, used for 7 days during T4 treatment from P15 to 21 (see Methodological Considerations in the Discussion for further details). All MMI-treated rats received 1% KClO_4_ with the MMI drinking solution until P21 in order to inhibit thyroidal iodine uptake and thyroid hormone synthesis during fetal and lactating periods. In the text, MMI_*P*__0__–__21_ and MMI_*P*__0__–__21_ + T4_*P*__15__–__21_ groups are referred to collectively as transient hypothyroid rats, and individually when significant differences between these two groups were found. Similarly, MMI_*P*__0_ and MMI_*E*__10_ groups are referred to as chronic hypothyroid rats. Control (C) rats were sacrificed at P150, P180 and P365. Four litters per group were used and culled to 8 pups per litter. After weaning, dams and female pups were anesthetized by 1.5–2% isoflurane inhalation (Laboratorios Dr. Esteve, S.A., Barcelona, Spain) in O_2_ (0.9 L/min) and decapitated.

### Determination of Total Triiodothyronine (tT3) and tT4 Concentrations in Plasma

Using isoflurane anaesthesia, blood samples (∼1 ml) were taken from the heart ventricle using a heparinized syringe, at P15, P21, and P50 (8 rats/group; 2 per litter). Blood was centrifuged and plasma kept at –20°C. After extraction and purification of the plasma samples, total thyroxine and tri-iodothyronine plasmatic concentrations were obtained by radioimmunoassay (see details in Lucia et al., 2018).

### Electron Microscopy

The general procedure for our EM study has been previously described in detail (Lucia et al., 2018). Isoflurane anaesthetized rats at P150 (4 rats/group; 1 per litter) were perfused with saline and fixed with 4% paraformaldehyde, 1% glutaraldehyde, 0.1M sucrose, 0.002% CaCl_2_ in 0.1M phosphate buffer (pH:7.3–7.4; PB) for 10 min. The heads were kept at 4°C during 2 h, the brains were then carefully removed and postfixed overnight, at 4°C.

The brains were sectioned into 250 μm slices in the mid-sagittal plane using a vibratome to obtain transversally cut callosal axons. From each hemisphere the two most central slices were OsO_4_ postfixed (30 min at room temperature) and uranyl acetate stained (1 h at 4°C in the dark), ethanol dehydrated, immersed in propylene oxide (Lab Baker, Deventry, Holland) and embedded overnight in Araldite (Durcupan, Fluka, Buchs SG, Switzerland). Semithin toluidine blue-stained sections (1.5 μm thick) and lead citrate-contrasted ultrathin sections (60–70 nm thin) were obtained using an ultra-microtome (Ultracut UC-6, Leica, Heidelberg, Germany). Images were obtained with a transmission electron microscope (FEI Tecnai Spirit G2, Eindhoven, Netherlands), equipped with a digital camera (Morada, Soft Imaging System, Olympus). Tissue sections and perfused brains not used were stored in 0.05% sodium azide in PB at 4°C, for future studies.

**FIGURE 2:**
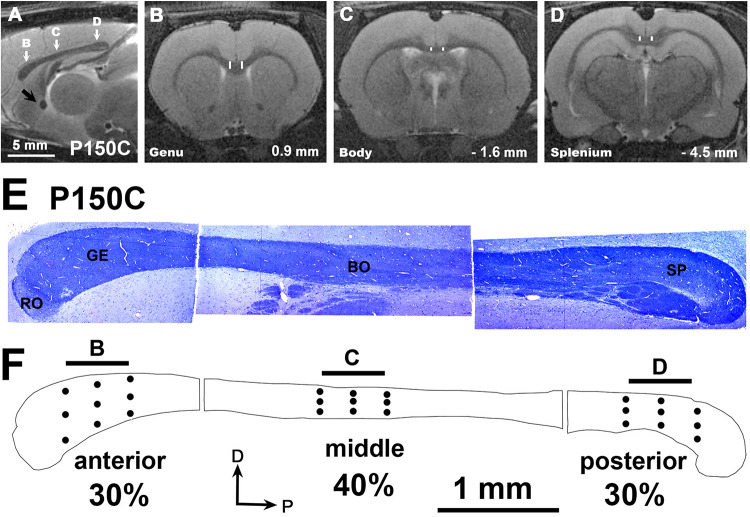
MRI images of a C rat at P150. **(A)** Mid parasagittal T2w image showing the antero-posterior locations (white arrows) from which the anterior **(B)**, middle **(C)** and posterior **(D)** coronal T2w images of were obtained. **(B–D)** Coronal images show the regions of interest (ROI) as the area between vertical white bars of the anterior, middle and posterior CC zones. Distances from Bregma are indicated in the lower-right corner, according to Paxinos et al. (2015). **(E)** Semithin toluidine blue-stained sections (1.5 μm thick) showing the structure of the CC at P150, and the main callosal regions: rostrum (RO), genu (GE), body (BO), and splenium (SP). Note that neither the isthmus nor the anterior and posterior zones of the body can be discerned, given that this region is mostly occupied by somatosensory axons in rodents. **(F)** Outlines show the anterior, middle and posterior zones comprising 30, 40, and 30 percent respectively of the total CC area. Dots give an approximate indication of where the EM micrographs and the T2w scans were taken (arrows in **A**). Horizontal bars (500 μm) at the top of the CC outline have the same length as the voxel z-thickness of T2w images of figures **(B–D)**. Same scale for figures **(A–D)** and for **(E,F)**.

The mid-sagittal transversal area of CC was obtained from outlines obtained by combining photographs from semithin sections (Figures 2E,F), and 30, 40, and 30% which was divided into three zones: the anterior 30% containing the genu, a middle 40% containing the body and the posterior 30% containing the splenium (Figure 2F).

Evenly spaced EM photomicrographs (16,500× magnification) were taken of the anterior, middle and posterior zones of each CC (9 photomicrographs on each zone; Figure 2F, dots). Unmyelinated and myelinated axon data, such as density, inner diameter and myelin thickness were obtained using the Cellgraph system (Microptic S.L., Barcelona, Spain). Unmyelinated and myelinated axon number in anterior, middle and posterior zones was calculated from mean axon density (measured in EM micrographs) and the corresponding mid-parasagittal area (measured in semithin sections). The outer diameter of myelinated axons was calculated as the sum of the axon inner diameter and 2 times its myelin thickness (see details in Berbel et al., 1994; Lucia et al., 2018). For myelinated axons, the g-ratio, and the conduction velocity (5.5 times the outer diameter) were calculated accordingly (Sanders and Whitteridge, 1946; Waxman and Bennett, 1972; Caminiti et al., 2013; Innocenti, 2017).

### MRI Data Acquisition and Processing

The MRI data acquisition parameters and images analyzed here are the same as those obtained for our previous study (Lucia et al., 2018). However in this study specific acquisition planes and region of interest (ROI) have been redefined, and fully described below. In summary, for MRI data acquisition, isoflurane anesthetized rats were scanned using a custom-made MRI compatible holder, positioned on the magnet isocenter, and monitored using a MRI compatible control unit (MultiSens Signal conditioner, OpSens, Quebec, Canada). Rats were maintained alive throughout the study period, with scans being taken periodically from P8 onward (Figure 1). Scans were obtained with a horizontal 7 Tesla scanner with a 30?cm diameter bore (Biospec 70/30v; Bruker Medical, Ettlingen, Germany), equipped with a 675 mT/m actively shielded gradient coil (Bruker Medical; BGA 12-S) of 11.4?cm inner diameter. A ^1^H rat brain receive-only phase array coil with integrated combiner and preamplifier (no tune/no match) was used, in combination with an actively detuned transmit-only resonator (Bruker BioSpin MRI) and Paravision software (Bruker Medical).

The coronal and sagittal planes of study were determined from preliminary T2-weighted (T2w) images acquired in the three orthogonal planes using rapid acquisition relaxation enhanced sequence (RARE) with the following parameters: RARE factor 8, 15 slices, slice thickness 1 mm, field of view (FOV) 40 mm × 40 mm, matrix 256 × 256, effective echo time (TE_*eff*_) 56 ms, repetition time (TR) 2,000 ms, 1 average for 1 min 4 s total acquisition time (Hennig and Friedburg, 1988; Perez-Cervera et al., 2018). Using these anatomical images, final coronal MRI images were acquired using RARE sequence with the following parameters: RARE factor 8, 25 slices, slice thickness 0.5 mm, FOV 20 mm × 20 mm, matrix 200 × 200 (voxel size 100 μm × 100 μm × 500 μm), TE_*eff*_ 56 ms, TR 3,728 ms, 4 averages for 12 min 26 s total acquisition time (Lucia et al., 2018).

MRI images were obtained from 8 rats per group (2 rats per litter) at ages from P8 to P365, with the rats of each group individually scanned at the ages indicated in Figure 1 and images analyzed using the ImageJ software (National Institute of Health, Bethesda, MD, United States). The ROI included the central portion at ± 0.7 mm from the midline in the anterior (located in the genu), middle (located in the body) and posterior (located in the splenium) zones of the CC (Figures 2A–D, vertical white lines in Figures 2B–D). T_2_r was calculated as the ratio between the signal intensity of T2w in the ROI to that of T2w in a ROI of the lateral ventricle cerebrospinal fluid. T_2_r was obtained in order to compare T2w signals between callosal zone, age and experimental groups. During postnatal development, T2w values of the lateral ventricle were similar between MMI and C rats (mean T2w values were 12,565 ± 1242 in transient and 12,161 ± 1146 in chronic hypothyroid, and 12,327 ± 961 in C rats; all T2w values ranging from 10,431 to 14,518).

Due to brain shrinkage found in MMI rats, anterior, middle and posterior anatomical landmarks were used to identify MMI and C sections located at equivalent anterio-posterior planes. In MMI and C rats at P150, the anterior landmark was the point where the optic nerves begin to form the optic chiasm (at 0.9 mm from Bregma in C rats; Paxinos et al., 2015); the middle landmark was the point where the rostral hippocampus begins to appear (at -1.6 mm from Bregma in C rats); and the posterior landmark was the caudal end-point of the hippocampal commissure (at -4.5 mm from Bregma in C rats; Figures 2B–D). At these levels, the motor, somatosensory, auditory and visual cortices were present (Supplementary Figure S1). In MRI scans at P150, contralateral distances between four selected homotopic contralateral cortical areas projecting through the CC were measured in four rats per group, specifically the projections of anterior (motor), middle (somatosensory), and posterior (auditory and visual) cortices. Contralateral distances were measured between mid-zones of pial surface in the selected neocortical areas (Supplementary Figure S1, white dots). As in the anterior commissure (Lucia et al., 2018), these measurements have a potential antero-posterior error of about 250 μm per hemisphere, given the MRI scan thickness of 500 μm. An estimation of myelinated axon conduction delay between selected homotopic areas was calculated from conduction velocity and mean contralateral distance between homotopic areas (Supplementary Figure S3 and Supplementary Table S6).

### Statistical Analysis

For statistical analysis (see details in Lucia et al., 2018), we used SYSTAT software (Systat Software, Inc., Chicago, IL, United States). Two-way ANOVA followed by either Tukey’s (equal variances) or Games-Howell’s (unequal variances) tests to identify significant differences (*P* ≤ 0.05) between means among age and experimental groups were used for mean frequency distribution analysis of MRI and EM data. One-way ANOVA followed by either Tukey’s test or the Student-Newman-Keuls method was used for the analysis of plasma concentration of thyroid hormones. The coefficient of determination (*R*^2^) calculated from non-linear and linear regression functions, provides a measure of how well estimated values correspond with observed values.

## Results

### Body Weight and Thyroid Hormone Levels

**FIGURE 3:**
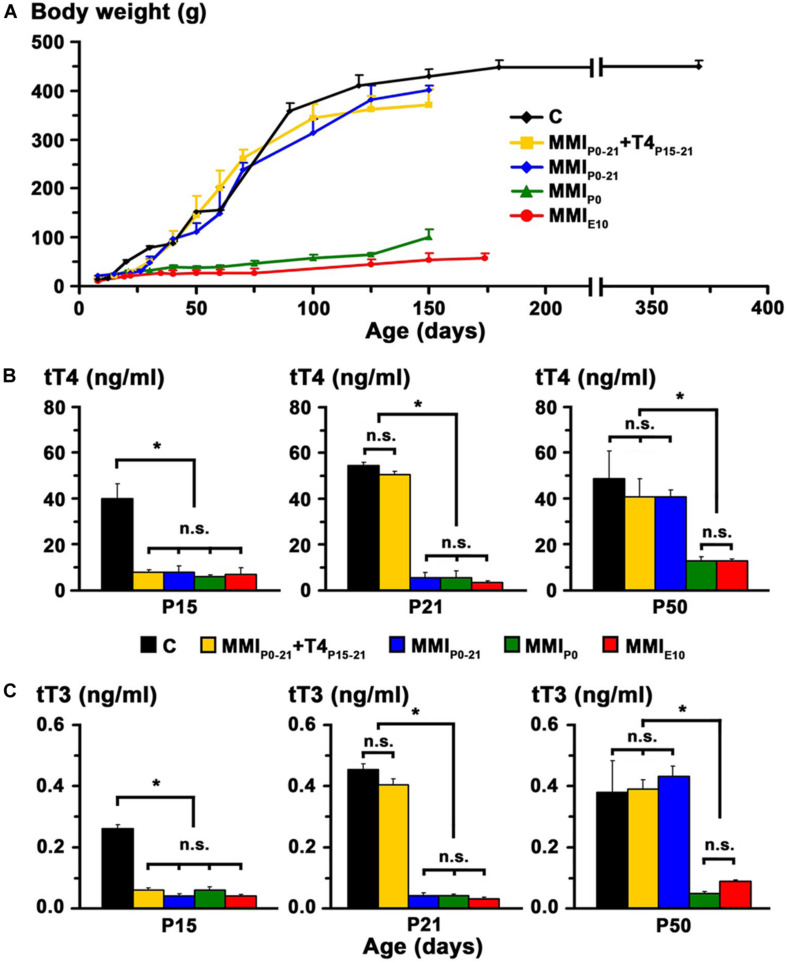
Body weight and plasma concentration levels of thyroid hormone. **(A)** Changes in body weight with age for C and MMI rats. Note the arrested growth of chronic hypothyroid rats. **(B,C)** Bar charts show the total plasma concentrations of T4 (tT4) and T3 (tT3) at the ages indicated. TH plasma concentrations were recovered in transient hypothyroid rats at P50. Bars: mean ± SD. n.s., non-significant differences. Significant differences: *P < 0.001 (*n* = 8–11 rats per group). Data and figure legend from Lucia et al. (2018).

In transient hypothyroid and C rats, body weight rapidly increased to 400 ± 20 g by P150 and then more slowly to 446 ± 13 g in C rats at P365. Weight was significantly lower (*P* < 0.001) in chronic hypothyroid rats with an average value of 75 ± 25 gr at P150 (Figure 3A).

At P15, plasma concentration levels of total T4 (tT4) and total T3 (tT3) in MMI rats had an average value of 7.2 and 0.05 ng/ml respectively, and were significantly lower (*P* < 0.001) than the control averages of 40.1 ng tT4/ml and 0.26 ng tT3/ml. Normal levels of tT4 and tT3 were reached at P21 in MMI_*P*__0__–__21_ + T4_*P*__15__–__21_ rats and at P50 in MMI_*P*__0__–__21_ rats. Levels of tT4 and tT3 in P50 chronic hypothyroid rats were significantly lower (*P* < 0.001) than in transient hypothyroid and C rats of the same age (Figures 3B,C).

As already mentioned, these data correspond to rats used in a previous study (Lucia et al., 2018).

### MRI Data

**FIGURE 4:**
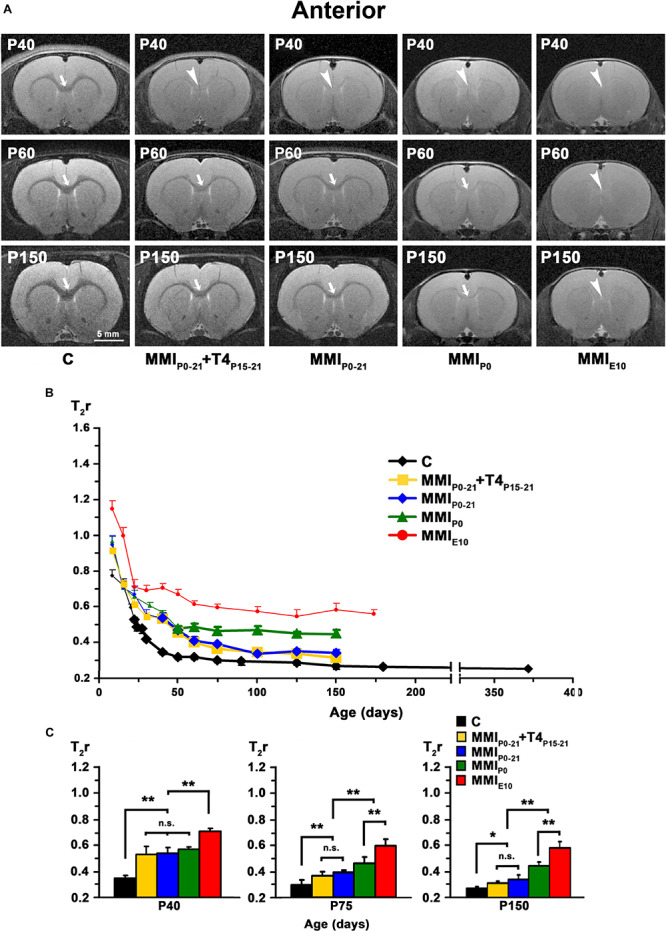
MRI images and T_2_r of the anterior postnatal CC. **(A)** At all ages, T_2_-w images of anterior CC (arrows and arrowheads) in MMI rats were less contrasted than in controls (arrowheads point to an undistinguishable anterior CC). The CC is hardly visible in MMI_*P*__0_ rats at P150 (arrow). Note the decreased contrast of CC in transient hypothyroid (MMI_*P*__0__–__*P*__21_ + T4_*P*__15__–__*P*__21_ and MMI_*P*__0__–__*P*__21_) rats at P40. **(B)** Graph showing T_2_r at postnatal ages. Bold symbols indicate that anterior CC was darker than the adjacent neuropil. In C rats, T_2_r decreased rapidly from P8 to P40 and then more slowly. In MMI rats, T_2_r values followed a similar trend but maintained higher values. **(C)** Bar charts show that T_2_r was significantly higher in MMI than in C rats at all ages. At P40, differences between transient and MMI_*P*__0_ rats were not significant, but significant differences were seen between these groups and MMI_*E*__10_ and C rats (*P* < 0.001). At P75, significant T_2_r differences (*P* < 0.001) were found between transient hypothyroid and both chronic (MMI_*P*__0_ and MMI_*E*__10_) and C rats. At P150, transient hypothyroid values were still significantly different to chronic hypothyroid (*P* < 0.001) and C (*P* < 0.05) rats. At all ages, significant differences between MMI_*P*__0_ and MMI_*E*__10_ (*P* < 0.001) rats were found. Bars: mean ± SD. n.s., non-significant differences. Significant differences: *P ≤ 0.05 and **P ≤ 0.001 (*n* = 8 rats per group). All figures at same scale.

**FIGURE 5:**
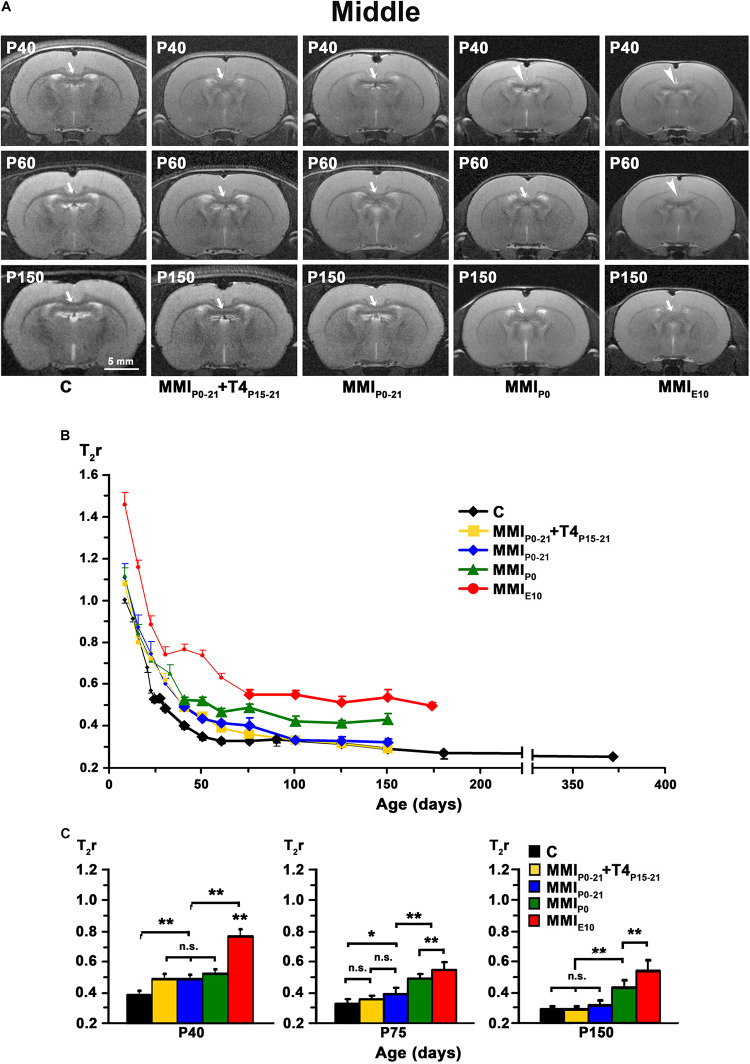
MRI images and T_2_r of the middle postnatal CC. **(A)** At all ages, T_2_-w images of the middle (arrows and arrowheads) of MMI rats were less contrasted than in controls (arrowheads point to an undistinguishable middle CC). The CC is hardly visible in chronic hypothyroid (MMI_*P*__0_ and MMI_*E*__10_) rats at P40 and P60, respectively. Note the decreased contrast of the body in transient hypothyroid (MMI_*P*__0__–__21_ and MMI_*P*__0__–__21_ + T4_*P*__15__–__21_) rats at P40. **(B)** Graph showing T_2_r at postnatal ages. Bold symbols indicate that anterior CC was darker than the adjacent neuropil. In C rats, T_2_r decreased rapidly from P8 to P60 and then more slowly. In MMI rats, values followed a similar trend but maintained higher values. **(C)** Bar charts show that T_2_r was significantly higher (*P* < 0.001) in chronic hypothyroid than in C rats at all ages. At P40, Differences between transient and MMI_*P*__0_ rats were not significant, but transient rats were significantly different (*P* < 0.001) to chronic hypothyroid and C rats. At P 75, differences (*P* < 0.001) were found between transient and chronic hypothyroid rats. The difference between MMI_*P*__0__–__21_ and C rats also significant (*P* < 0.05). At P150, differences between transient and C rats were not significant, however both transient and chronic hypothyroid were significantly different (*P* < 0.001) to C rats. At all ages, significant differences between MMI_*P*__0_ and MMI_*E*__10_ (*P* < 0.001) rats were found. Bars: mean ± SD. n.s., non-significant differences. Significant differences: *P ≤ 0.05 and **P ≤ 0.001 (*n* = 8 rats per group). All figures at same scale.

**FIGURE 6:**
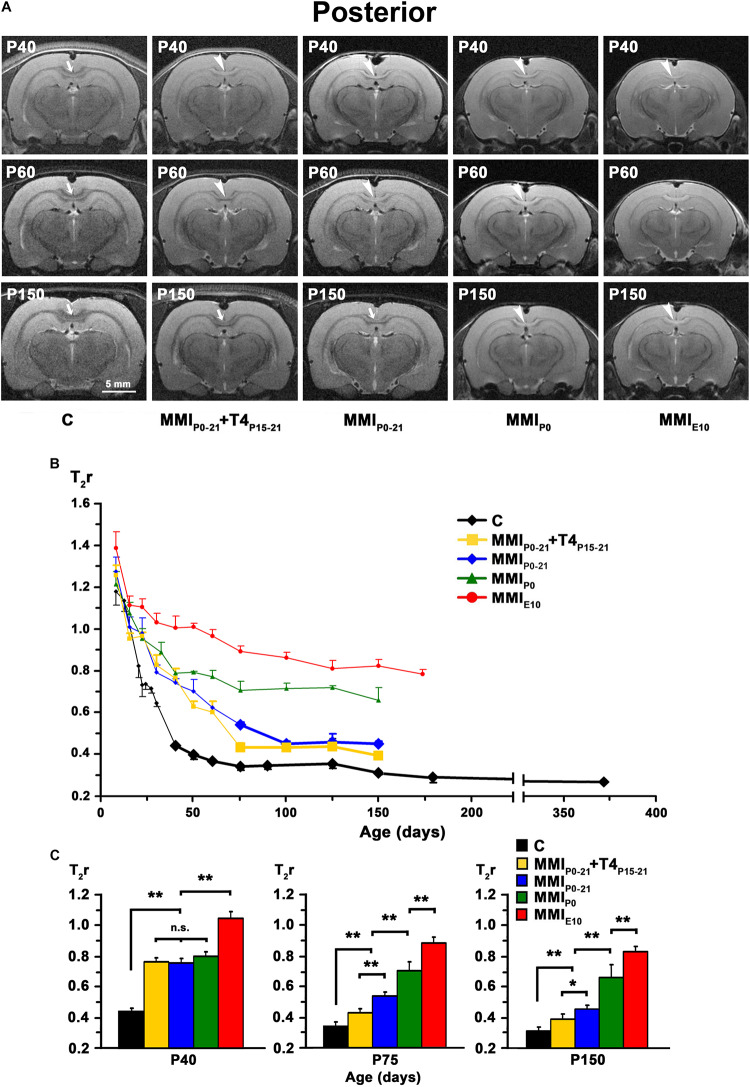
MRI images and T_2_r of the posterior postnatal CC. **(A)** At all ages, T_2_-w images of the posterior CC (arrows and arrowheads) of MMI rats were less contrasted than in controls (arrowheads point to an undistinguishable posterior CC). The CC is hardly visible in chronic hypothyroid (MMI_*P*__0_ and MMI_*E*__10_) rats at P150. Note the decreased contrast of the posterior CC in transient hypothyroid (MMI_*P*__0__–__21_ + T4_*P*__15__–__*P*__21_ and MMI_*P*__0__–__21_) rats at P150. **(B)** Graph showing T_2_r at postnatal ages. Bold symbols indicate that the posterior CC was darker than the adjacent neuropil. In C rats, T_2_r decreased rapidly from P8 to P75 and then more slowly. In MMI rats, values followed a similar trend but maintained higher values. **(C)** Bar charts show that T_2_r was significantly higher (*P* < 0.001) in chronic hypothyroid than in C rats at all ages. At P40, Differences between transient and MMI_*P*__0_ rats were not significant, but significantly different (*P* < 0.001) to chronic hypothyroid and C rats. At P75 and P150, significant differences (*P* < 0.001) were found between all groups of MMI treated rats and controls, except for a decrease in difference (*P* < 0.05) between untreated and T4-treated transient hypothyroid rats at P150. At all ages, significant differences between MMI_*P*__0_ and MMI_*E*__10_ (*P* < 0.001) rats were found. Bars: mean ± SD. n.s., non-significant differences. Significant differences: *P ≤ 0.05 and **P ≤ 0.001 (*n* = 8 rats per group). All figures at same scale.

At early ages, anterior, middle and posterior CC appeared lighter and hardly distinguishable from the adjacent neuropil in T2w images from both MMI and C rats. Anterior CC showed increased border definition in C rats at P30, and in middle and posterior CC at P40 (arrows in Figures 4A, 5A, 6A). Anterior and middle CC remained hardly distinguishable in MMI_*P*__0_ at P40 and in MMI_*E*__10_ rats at P60 and P150 (see arrowheads in Figures 4A, 5A), as did posterior CC, in transient hypothyroid rats at P60 and in chronic hypothyroid rats at P150 (arrowheads in Figure 6A and Supplementary Tables S1–S3).

In C rats, T_2_r decreased rapidly from P8 (on average, T_2_r = 0.99 ± 0.21) to P40 in anterior (T_2_r = 0.35 ± 0.01), to P60 in middle (0.33 ± 0.01), and to P75 in posterior CC (0.34 ± 0.02). This was followed by a more slow decline, reaching T_2_r = 0.25 ± 0.01, 0.26 ± 0.01 and 0.27 ± 0.03, in anterior, middle and posterior CC respectively at P365. T_2_r in MMI rats also decreased rapidly from P8 to P40 but remained higher than controls (Figures 4B, 5B, 6B, Supplementary Figure S2, and Supplementary Tables S1–S3). Anterior CC at P40 and P75 showed significant T_2_r differences between MMI and C rats (*P* < 0.001). At P150, differences decreased (*P* < 0.05) between transient hypothyroid and C rats, but still remained higher (*P* < 0.001) in chronic hypothyroid (Figure 4C and Supplementary Table S1). In middle CC, significant T_2_r differences were found between MMI and C rats at P40 (*P* < 0.001). At P75, no differences were found between MMI_*P*__0__–__21_ + T4_*P*__15__–__21_ and C rats; and a significant difference (P < 0.05) was found between MMI_*P*__0__–__21_ and C rats. At P150, differences remained (*P* < 0.001) between chronic hypothyroid rats with respect to the other groups; and a significant difference (*P* < 0.05) was found between MMI_*P*__0__–__21_ and MMI_*P*__0__–__21_ rats (Figure 5C and Supplementary Table S2). In posterior CC, significant T_2_r differences (*P* < 0.01) were found between MMI and C rats at all ages (Figure 6C and Supplementary Table S3), and remained higher in transient and chronic hypothyroid rats in anterior and posterior CC (Supplementary Figure S2).

### EM Study

**FIGURE 7:**
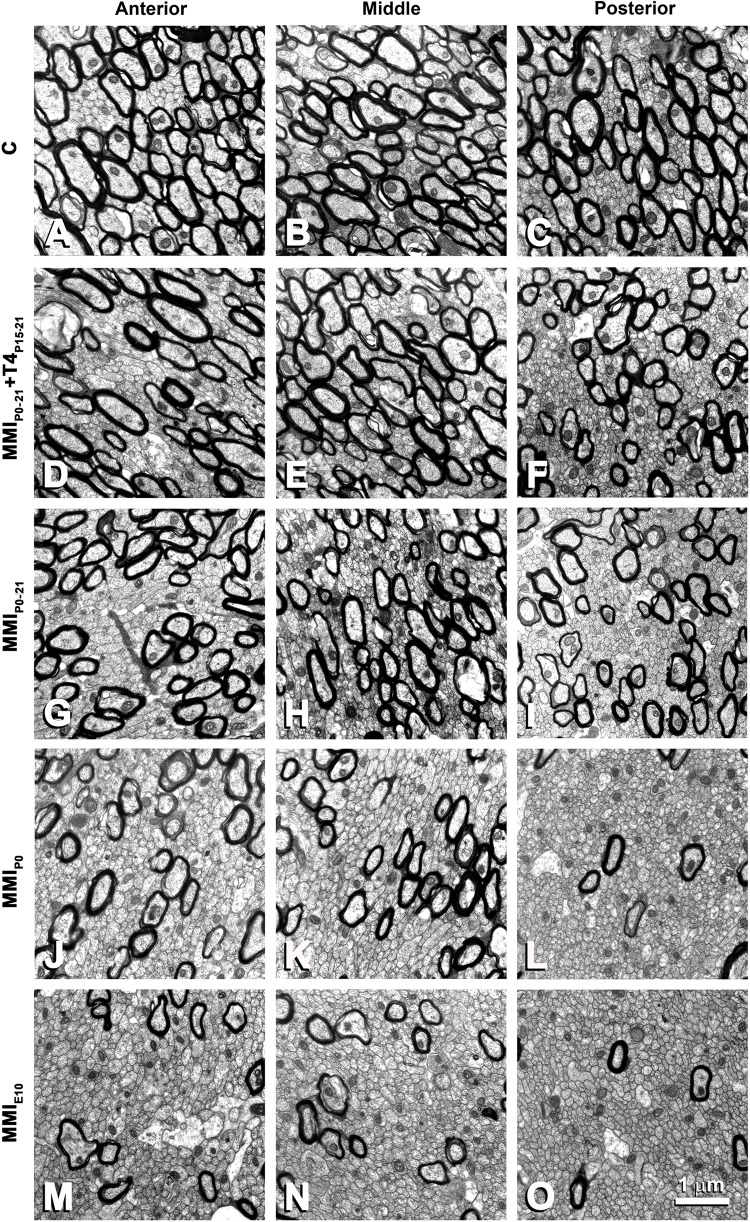
EM photomicrographs of the CC ultrastructure in MMI and C rats at P150 in anterior **(A,D,G,J,M)**, middle **(B,E,H,K,N)** and posterior **(C,F,I,L,O)** CC. Note the decreased posterior myelinated axon density, compared to anterior and middle CC in MMI and C rats. Compared to transient and C, the density of myelinated axons is significantly decreased in chronic MMI rats **(J–O)**. No differences in the myelin thickness can be seen. All figures at same scale.

The ultrastructure of commissural axons and glial processes in anterior, middle and posterior CC at P150 observed in this study (Figure 7) was similar to that previously described in C (Gravel et al., 1990; Berbel et al., 1994) and chronic hypothyroid MMI-treated rats (Berbel et al., 1994). The decreased myelinated axon density in anterior, middle and posterior CC of chronic hypothyroid (Figures 7J,O) is noteworthy compared to C (Figures 7A,C) rats. The CC mid-sagittal area was 2,539,483 ± 479,729 μm^2^ in C rats; it significantly decreased to 1,933,471 ± 195,946 μm^2^, on average, in transient hypothyroid (*P* < 0.01), and to 957,134 ± 10,480 μm^2^, on average, in chronic hypothyroid rats (*P* < 0.001), representing a 23.9 and 62.3% reduction, respectively compared to C rats (Supplementary Table S4).

**FIGURE 8:**
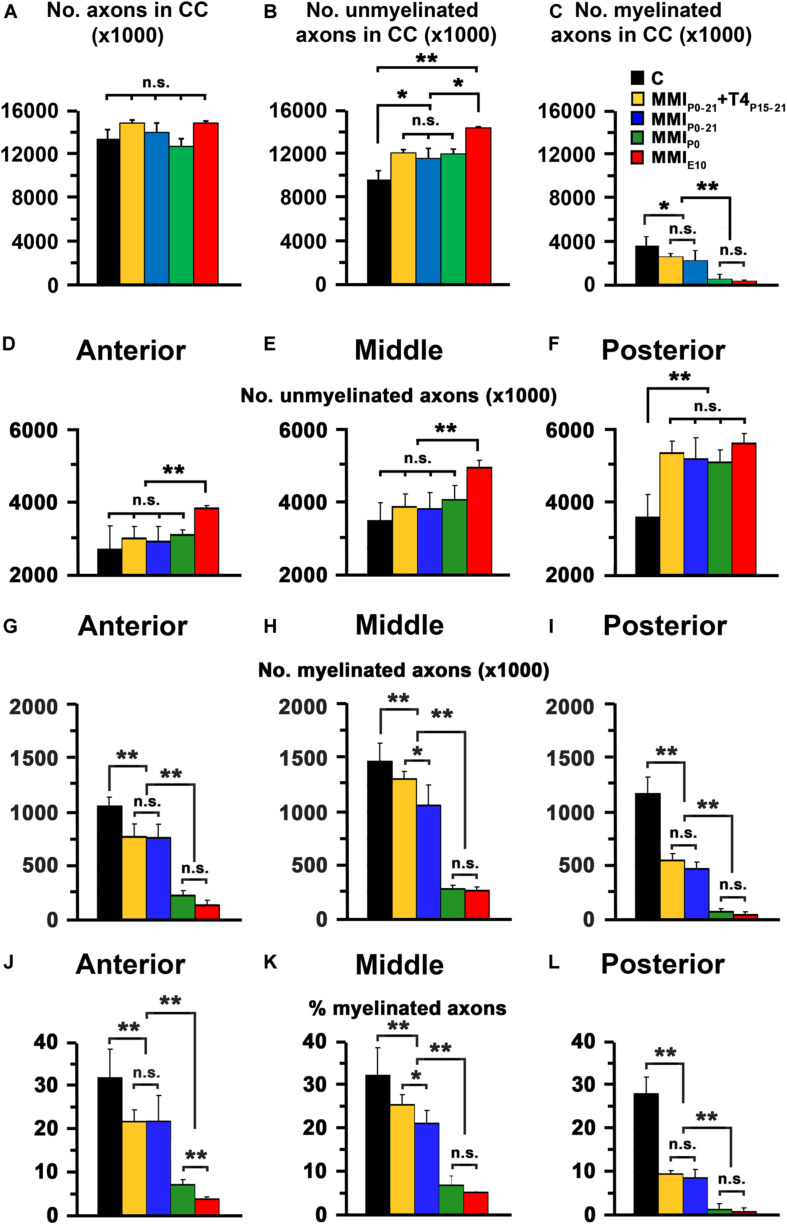
EM quantitative data of the CC at P150. **(A–C)** Bar charts show total, unmyelinated and myelinated axon number in the CC. Unmyelinated **(D–F)** and myelinated axon number **(G–I)**, and myelinated axon percentage **(J–L)** in the anterior, middle and posterior CC are shown. **(A)** Differences in the number of MMI and C axons were not significant. **(B)** The difference in unmyelinated axon number between transient (MMI_*P*__0__–__21_ + T4_*P*__15__–__*P*__21_ and MMI_*P*__0__–__21_) and MMI_*P*__0_ rats was not significant, transient and MMI_*P*__0_ numbers were significantly different (*P* < 0.05) to MMI_*E*__0_ chronic hypothyroid and C rats. **(C)** The number of myelinated axons of transient hypothyroid rats decreased significantly (*P* < 0.05) with respect to C rats and even more so in chronic hypothyroid rats (*P* < 0.001). **(D,E)** The unmyelinated axon number in anterior and middle CC in transient and MMI_*P*__0_ rats was not significantly different to C rats but increased significatively (*P* < 0.001) increased in MMI_*E*__10_ rats. **(F)** In the posterior CC, there was no significant difference in unmyelinated axon number between transient and hypothyroid rats which were both significantly higher (*P* < 0.001) compared to C rats. In contrast, myelinated axon number **(G–I)** and percentage **(J–L)** in the anterior, middle and posterior CC, decreased significantly in all MMI rats compared to controls, with the lowest values found in chronic hypothyroid rats. In middle CC **(H,K)**, significant differences (*P* < 0.05) were found between untreated and T4-treated transient hypothyroid rats. Errors bars: SD. n.s., non-significant differences. Significant differences: *P ≤ 0.05 and **P ≤ 0.001 (*n* = 4 rats per group).

In agreement with previous data (Berbel et al., 1994), the total axon number in the CC was similar in C and MMI rats at P150, ranging from 12,706,226 axons in MMI_*P*__0_ to 14,913,049 axons in MMI_*E*__10_ rats (13,362,275 in C rats) (Figure 8A and Supplementary Table S4). Compared to C rats, unmyelinated CC axon number significantly increased in transient hypothyroid and MMI_*P*__0_ (*P* < 0.05), and in MMI_*E*__10_ (*P* < 0.001) rats (Figure 8B), while myelinated axon number decreased in transient (*P* < 0.05) and in chronic (*P* < 0.001) hypothyroid rats (Figure 8C). In anterior and middle CC, unmyelinated axon number remained higher in MMI_*E*__10_ (*P* < 0.001; Figures 8D,E) and in posterior CC, in MMI rats (*P* < 0.001; Figure 8F than in other groups. In anterior, middle and posterior CC, myelinated axon number decreased significantly (*P* < 0.001) in transient and chronic hypothyroid rats compared to C rats (Figures 8G–I and Supplementary Table S4). In transient hypothyroid rats, the average decrease of the myelinated axon was 29.7% in anterior and middle CC and 67.7% in posterior CC compared to C rats. In chronic hypothyroid rats, it was 82.5% in the anterior and middle CC and 96.0% in the posterior CC compared to C rats (Figures 8J–L and Supplementary Table S4).

**FIGURE 9:**
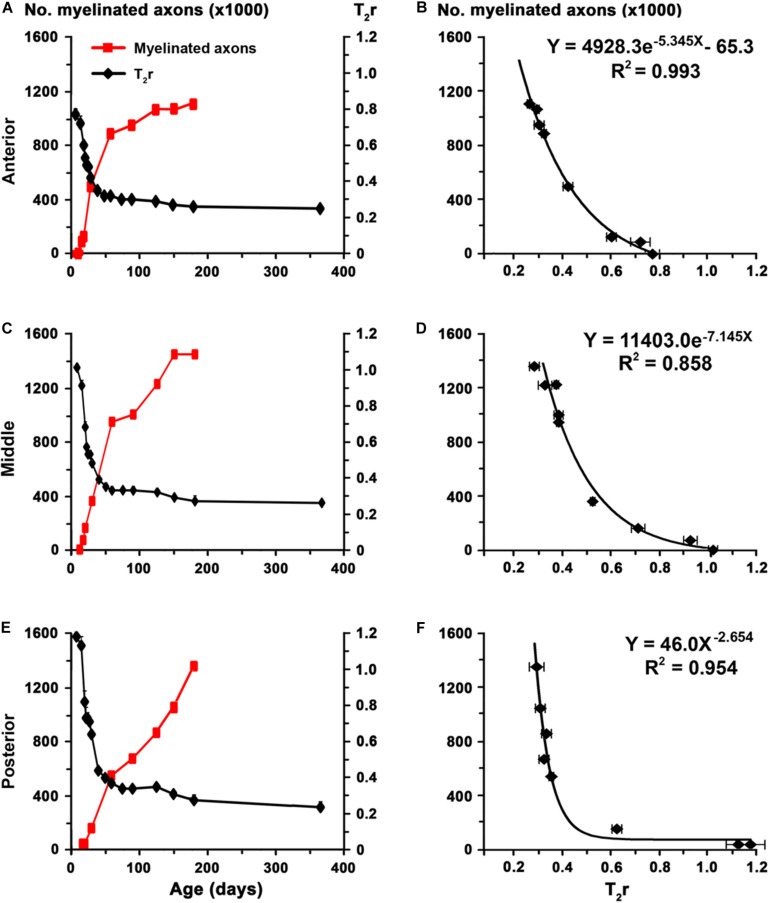
Regression functions between EM and T_2_r values in the postnatal CC. **(A,C,E)** Graphs show T_2_r (black lines) and myelinated axon number (red lines; data from Berbel et al., 1994) in anterior, middle and posterior CC in postnatal C rats at different ages. **(B,D,F)** Regression functions between myelinated axon number and T_2_r. High fits were observed in the anterior (**B**; *R*^2^ = 0.993), middle (**D**; *R*^2^ = 0.858), and posterior (**F**; *R*^2^ = 0.954) CC.

**FIGURE 10:**
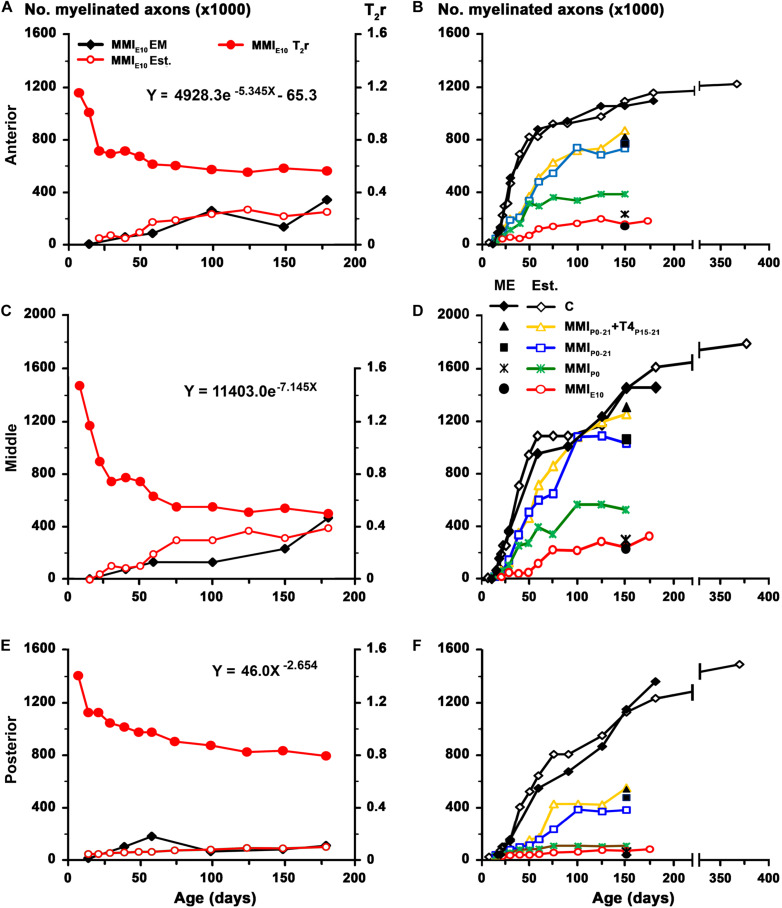
Estimated values for MMI treated rats. Estimated values were calculated using the paradigm published for work on the anterior commissure (Lucia et al., 2018). To assess the regression functions from Figures 9B,D,F, estimated values of myelinated axon number (white fill red circles) in the anterior **(A)**, middle **(C)**, and posterior **(E)** CC for MMI_*E*__10_ rats were obtained using T_2_r values (solid red circle) and plotted Note that estimated values were similar to those previously published in EM studies (solid black diamond; data from Berbel et al., 1994). These regression functions were used to estimate myelinated axon number in the anterior **(B)**, middle **(D)**, and posterior **(F)** CC at postnatal ages in C, transient and chronic hypothyroid rats. At P150, estimated values of myelinated axon number of C rats (white fill diamond) were similar to published values (solid black diamond; data from Berbel et al., 1994). Estimated values for myelinated axon number in anterior CC decreased **(B)** in transient hypothyroid (on average, 23.2%), MMI_*P*__0_ (63.8%), and MMI_*E*__10_ (79.9%) rats compared to C. A similar decrease was found in middle **(D)** and posterior **(F)** CC. At P150, the estimated number of myelinated axons for transient and chronic hypothyroid rats was similar to the number of myelinated axons found in ME, except for MMI_*P*__0_ rats (filled black symbols; **B,D,F**). Est., estimated values.

The biological significance of MRI in the development of the CC was explored by generating the corresponding regression function between T_2_r and quantitative EM data. Using the EM data from the CC in C rats at P150 and at different postnatal ages (Berbel et al., 1994), we found a significant fit between T_2_r and myelinated axon number in the anterior (*R*^2^ = 0.993; Figures 9A,B), middle (*R*^2^ = 0.858; Figures 9C,D) and posterior CC (*R*^2^ = 0.954; Figures 9E,F). No fit was found between T_2_r and other quantitative EM data such as myelin thickness (*R*^2^ = 0.582). The regression functions between T_2_r and myelinated axon number in the three regions of the CC were validated by comparing the estimated myelinated axon number for MMI_*E*__10_ rats with values obtained from already published EM data (MMI_*E*__10_ group in Berbel et al., 1994). No significant differences were found between estimated MMI_*E*__10_ values and those published (Figures 10A,C,E). Regression functions were used to compare the estimated myelinated axon number for C, transient and chronic hypothyroid rats at different postnatal ages, and P150 values were compared with the EM values. Estimated and EM values were similar, except for MMI_*P*__0_ anterior (233,500 axons vs. 378,900 estimated axons) and middle CC (300,300 axons vs. 528,100 estimated axons; Figures 10B,D,F and Supplementary Table S4).

### Axon Measurements, g-Ratio, and Estimation of Conduction Velocity

**FIGURE 11:**
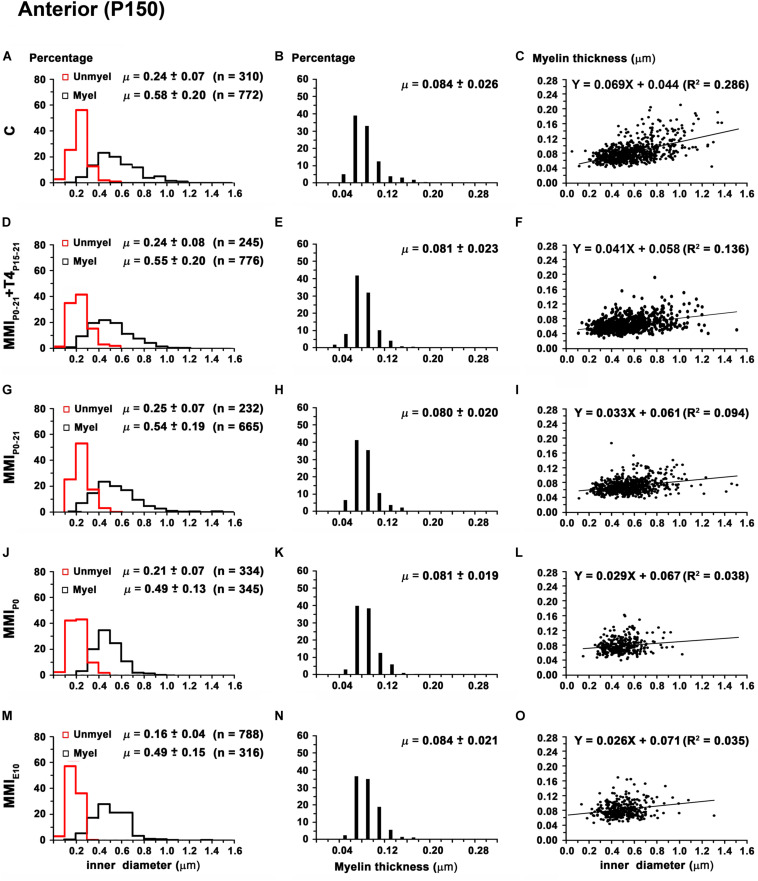
Distribution of myelinated axon diameter and myelin thickness in the anterior CC at P150. **(A,D,G,J,M)** Histograms show unmyelinated and myelinated axon diameter distributions. Mean unmyelinated and myelinated axon diameter decreased (*P* < 0.001) in chronic hypothyroid rats compared to transient and C rats. **(B,E,H,K,N)** Histograms show myelin thickness distributions. The mean myelin thickness was similar between MMI and C rats. **(C,F,I,L,O)** Plots show the association between myelinated axon inner diameter and myelin thickness. Note the poor fit found between groups (*R*^2^ range: 0.035–0.286), however the slope of the regression function was higher in C (3.9°) than in MMI (on average, 1.8°) rats. Unmyel, unmyelinated. Myel, myelinated. *R*^2^, determination coefficients. n, number of axons and means (μ = mean ± SD) are indicated.

**FIGURE 12:**
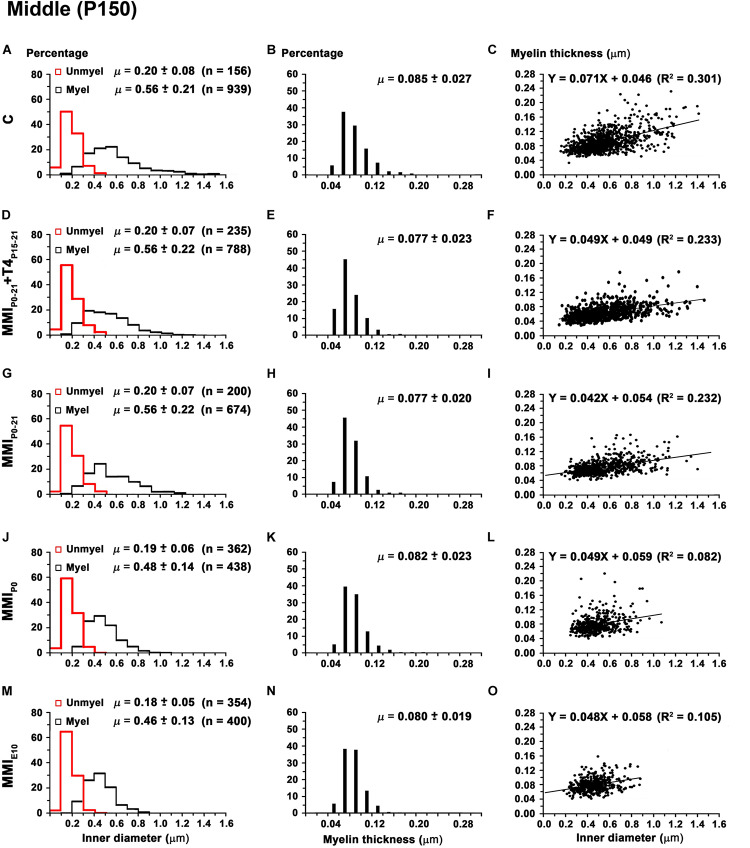
Distribution of myelinated axon diameter and myelin thickness in the middle CC at P150. **(A,D,G,J,M)** Histograms show unmyelinated and myelinated axon diameter distributions. Mean unmyelinated and myelinated axon diameter decreased (*P* < 0.001) in chronic hypothyroid rats compared to transient and C rats. **(B,E,H,K,N)** Histograms show myelin thickness distributions. The mean myelin thickness was similar between MMI and C rats. **(C,F,I,L,O)** Plots show the association between myelinated axon inner diameter and myelin thickness. Note the poor fit found between groups (*R*^2^ range: 0.082–0.301), however the slope of the regression function was higher in C (4.1°) than in MMI (on average, 2.7°). Unmyel, unmyelinated. Myel, myelinated. *R*^2^, determination coefficients. n, number of axons and means (μ = mean ± SD) are indicated.

**FIGURE 13:**
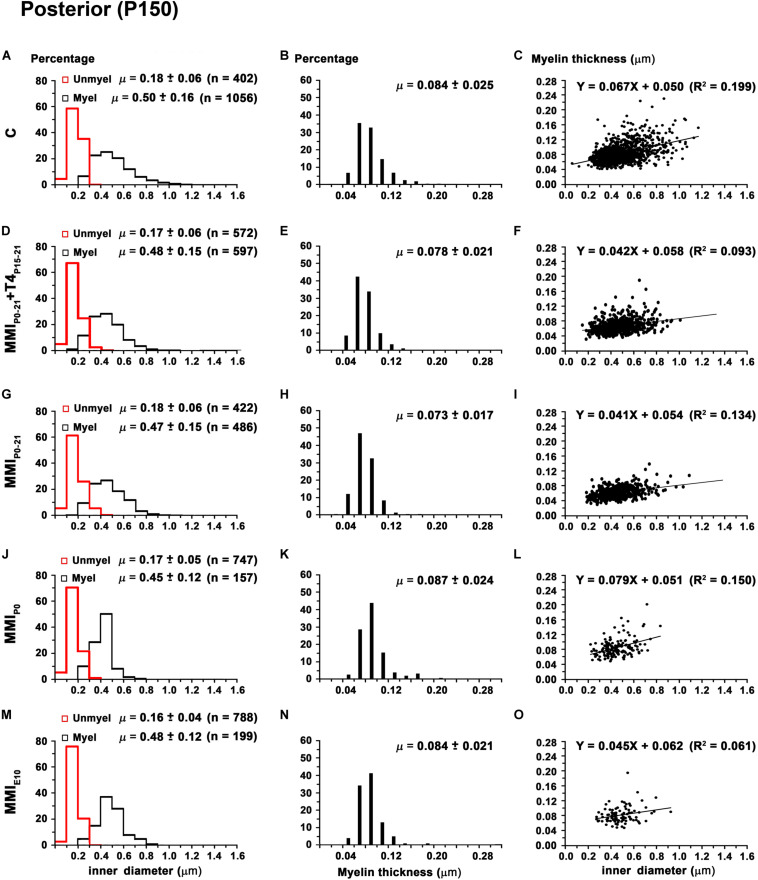
Distribution of myelinated axon diameter and myelin thickness in the posterior CC at P150. **(A,D,G,J,M)** Histograms show unmyelinated and myelinated axon diameter distributions. Mean unmyelinated and myelinated axon diameter decreased (*P* < 0.001) in chronic hypothyroid rats compared to transient and C rats. **(B,E,H,K,N)** Histograms show myelin thickness distributions. The mean myelin thickness was similar between MMI and C rats. **(C,F,I,L,O)** Plots show the association between myelinated axon inner diameter and myelin thickness. Note the poor fit found between groups (*R*^2^ range: 0.061–0.199), however the slope of the regression function was higher in C (3.8°) than in MMI (on average, 3.0°). Unmyel, unmyelinated. Myel, myelinated. μ = mean ± SD. *R*^2^, determination coefficients. n, number of axons.

Mean unmyelinated axon diameter decreased in posterior CC of all groups, except for MMI_*E*__10_ rats where posterior was similar to anterior, middle CC. The highest unmyelinated axon diameters were observed in anterior CC of C and transient hypothyroid rats (on average, 0.24 ± 0.07 μm) with MMI_*E*__10_ diameters being significantly lower (0.16 ± 0.04 μm; *P* < 0.001; Figures 11A,D,G,J,M). In middle and posterior CC, unmyelinated axon diameter was similar between groups Figures 12A,D,G,J,M, 13A,D,G,J,M and Supplementary Table S5).

In anterior and middle CC, myelinated axon inner diameter decreased (*P* < 0.001) in chronic hypothyroid rats (0.49 ± 0.14 and 0.47 ± 0.13 μm, respectively) compared to transient and C rats (on average, 0.56 ± 0.21 μm) (Figures 11A,D,G,J,M, 12A,D,G,J,M and Supplementary Table S5). In posterior CC, myelinated axon inner diameter decreased (*P* < 0.05) in transient and chronic (on average, 0.47 ± 0.13 μm) hypothyroid compared to C (0.50 ± 0.16 μm) rats (Figures 13A,D,G,J,M and Supplementary Table S5). Myelinated axons with an inner diameter ≥ 1.0 μm made up 8.6% in anterior, 7.9% in middle and 2.7% in posterior CC of C rats. A decreased inner diameter was found respectively in transient (3.8, 4.4 and 0.8%) and chronic hypothyroid (1.1, 0.3, and 0.0%) rats (Figures 11A,D,G,J,M, 12A,D,G,J,M, 13A,D,G,J,M).

Anterior CC myelin thickness (on average, 0.082 ± 0.022 μm) was similar throughout all groups (Figures 11B,E,H,K,N and Supplementary Table S5). Middle CC was significantly decreased (*P* < 0.01) in transient and chronic hypothyroid rats (on average, 0.078 ± 0.021 μm) compared to controls (0.085 ± 0.027 μm; Figures 12B,E,H,K,N and Supplementary Table S5). Posterior CC average myelin thickness was significantly lower (*P* < 0.01) in transient hypothyroid rats (0.075 ± 0.019 μm) compared to other groups (on average, 0.085 ± 0.022 μm; Figures 13B,E,H,K,N and Supplementary Table S5). No significant fit was found between myelin thickness and axon inner diameter in anterior, middle and posterior CC (*R*^2^ ranging from 0.061 to 0.199). Notwithstanding, the slope of the anterior, middle and posterior CC regression function was higher in C rats (3.9°, 4.1°, and 3.8°, respectively) than in transient (on average, 2.1°, 2.6°, and 2.4°) and chronic hypothyroid rats (on average, 1.6°, 2.8°, and 3.5°; Figures 11C,F,I,L,O, 12C,F,I,L,O, 13C,F,I,L,O).

**FIGURE 14:**
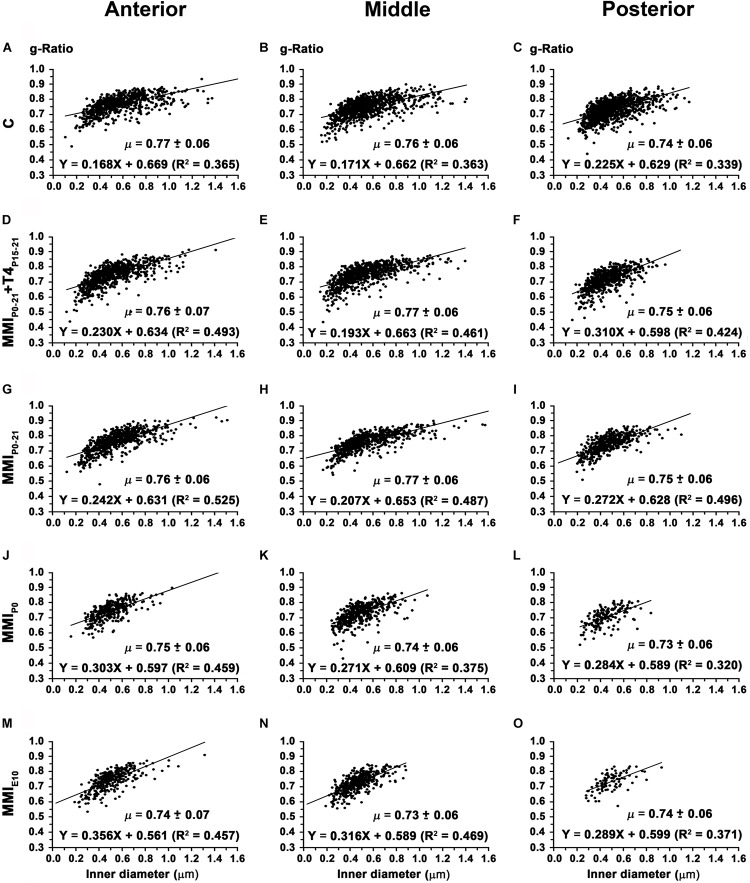
Association between axon inner diameter and g-ratio in the anterior, middle and posterior CC at P150. Plots show that in the anterior **(A,D,G,J,M)**, middle **(B,E,H,K,N)** and posterior **(C,F,I,L,O)** CC, the g-ratio was lower (*P* < 0.01) in chronic hypothyroid than in transient hypothyroid and C rats. g-Ratio ranges were from 0.74 to 0.77 in anterior, from 0.73 to 0.77 in middle and from 0.72 to 0.75 in posterior CC. μ = mean ± SD. *R*^2^, determination coefficients.

In anterior, middle and posterior CC, the average g-ratio (0.74 ± 0.06, 0.73 ± 0.06, and 0.73 ± 0.06, respectively) decreased significantly (*P* < 0.01) in chronic hypothyroid rats compared to transient hypothyroid and C rats (on average, 0.76 ± 0.06 in anterior, 0.77 ± 0.06 in middle and 0.75 ± 0.06 in posterior CC) (Figure 14 and Supplementary Table S5). The average slope of the regression functions for anterior, middle and posterior CC was lower in C (9.5°, 9.7°, and 12.7°) than in transient (13.3°, 11.3°, and 16.2°) and chronic (18.2°, 16.3°, and 16.0°) rats (Figure 14).

**FIGURE 15:**
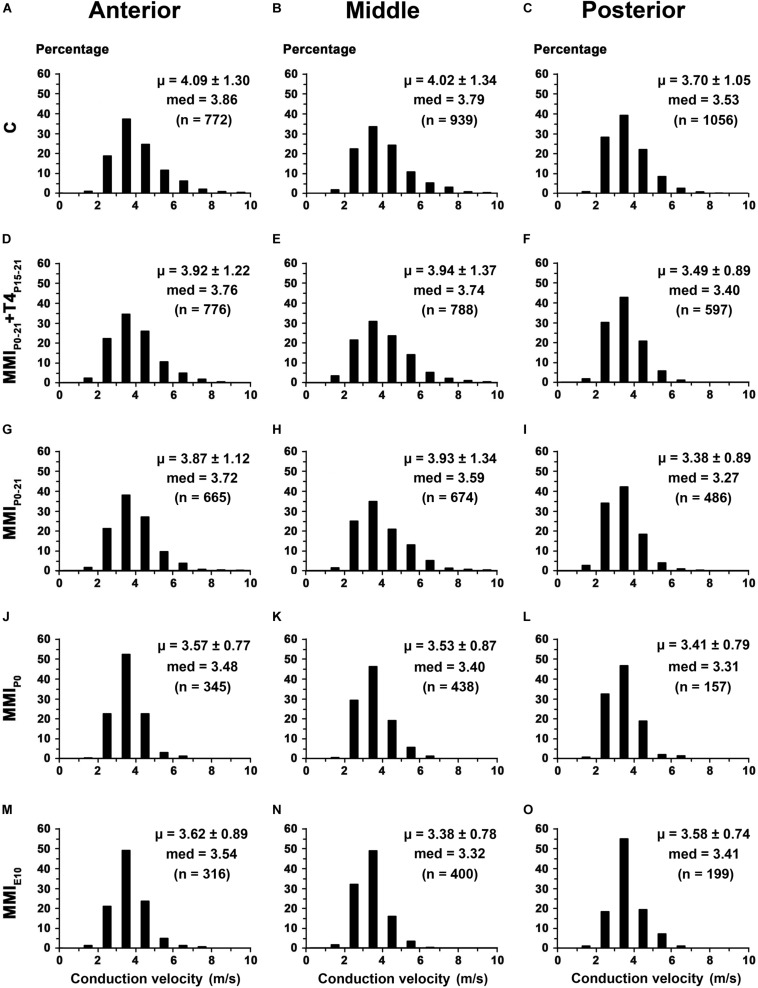
Distribution of myelinated axon conduction velocity of the CC in MMI and C rats at P150. **(A,D,G,J,M)** Histograms show myelinated axon conduction velocity distributions in anterior CC. Mean myelinated axon conduction velocity decreased in transient (*P* < 0.05) and chronic (*P* < 0.001) hypothyroid rats compared to C rats. **(B,E,H,K,N)** Histograms show myelinated axon conduction velocity distributions in middle CC. Mean myelinated axon conduction velocity decreased in chronic (*P* < 0.001) hypothyroid rats compared to transient and C rats. **(C,F,I,L,O)** Histograms show myelinated axon conduction velocity distributions in posterior CC. Mean myelinated axon conduction velocity decreased in transient and chronic (*P* < 0.01) hypothyroid rats compared to C rats. med, median. n, number of axons. μ = mean ± SD.

In anterior CC, the conduction velocity of myelinated axons decreased in transient (3.89 m/s; *P* < 0.05) and chronic (3.60 m/s; *P* < 0.001) hypothyroid rats compared to C rats (4.09 m/s; Supplementary Table S5). In middle CC, conduction velocity decreased in chronic hypothyroid rats (3.47 m/s) compared to the other groups (on average, 3.96 m/s; *P* < 0.001), while in posterior CC, it decreased in transient and chronic hypothyroid (3.47 m/s; *P* < 0.01) rats compared to C (3.70 m/s; Figure 15 and Supplementary Table S5). The average distance estimated between primary motor and sensory cortical areas decreased significantly in MMI rats (Supplementary Figures S1, S6 and Supplementary Table S6), commensurate with brain shrinkage (Figures 4A, 5A, 6A and Supplementary Figure S1). In summary, the estimated conduction delay between the homotopic cortical zones decreased in transient (range 5.3–16.8%, excluding auditory zones) and chronic (range 5.7–20.6%) hypothyroid rats when compared to controls (Supplementary Figure S6 and Supplementary Table S6).

## Discussion

Our main goal was to study the effect of early-postnatal transient hypothyroidism on CC development, using MRI and EM. Data were obtained from anterior, middle and posterior CC zones located respectively in the genu, body and splenium. These zones were chosen because they contain axons from the motor (genu), somatosensory (body), auditory and visual (splenium) cortical areas. Our results show that both postnatal transient and postnatal chronic hypothyroidism cause a greater decrease of myelinated axon number and percentage in posterior than in anterior and middle CC, and that this decrease is not recovered when T4-treatment is delayed. Regression functions, obtained from T_2_r values, show that estimated myelinated axon number in MMI rats at P150 is similar to the number found by direct axon counting in EM. In all MMI rats, mean unmyelinated and myelinated axon diameter decreases in the posterior CC, except in the case of MMI_*E*__10_ rats in which it remains low in all zones of the CC. g-Ratio is similar between MMI and C rats. The estimated conduction delays decrease in MMI rats, mostly due to shorter distances between homotopic callosal projecting areas.

### Methodological Considerations: Limitations and Strengths

The present study follows on from our previous study of the anterior commissure (Lucia et al., 2018), and has similar strengths and limitations. Its main strength is the combined use of non-invasive *in vivo* MRI analysis of the CC with an *ex vivo* EM qualitative and quantitative study of the number and diameter of unmyelinated and myelinated axons. Myelinated axon percentage could be estimate as a function of T_2_r values and was able to confirm altered CC postnatal development in late T4-treated transient hypothyroid rats.

The quantitative EM measures are based on average axon data from 36 samples on each CC region (4 rats and 9 sampling areas per rat). The values obtained for MMI_*E*__10_ and C rats at P150 were similar to those obtained in previous studies (Gravel et al., 1990; Berbel et al., 1994). Although the three antero-posterior regions aim to define callosal axons from motor, somatosensory, auditory and visual areas, different subpopulations of unmyelinated and myelinated axons are being averaged, and this could mask changes occurring in one or the other of these subpopulations. Further studies using electrophysiology, behavior and functional imaging, among other methodologies, will be needed to study identified subpopulations of callosal neurons.

The management of thyroid hormone concentrations administered to MMI pups from P15 to P21 is critical in the developing cerebral cortex because it is related to the amount of active hormone that target cells require for correct gene expression. Several events play a role such as: (i) postnatal angiogenesis which increases the surface for the transfer of thyroid hormones, (ii) the expression of thyroid hormone transporters carrying free thyroid hormones from serum to target neural cells, and (iii) the proliferation of type 2 deiodinase expressing astrocytes that mediate the deiodination of T4 to T3. Thyroid hormones levels in the cerebral cortex of early postnatal hypothyroid rats remain unknown. Despite the possible differences between males and females, the L-T4 dose administered in this study was adjusted from previously published data on 6 groups of young female rats thyroidectomized when weighing 120–150 g (approximate age P50–75) and 30 days later, subcutaneously infused with 6 different doses of L-T4 (using ALZET^®^ osmotic minipumps) spaced over 12–13 days with tT4 and tT3 being measured in plasma and different tissues, including the cerebral cortex (Escobar-Morreale et al., 1995). For our study, circulating tT4 and tT3 levels were measured at P15 and P21 (onset and end of L-T4 treatment). At P15, MMI_*P*__0__–__21_ + T4_*P*__15__–__21_ rats were hypothyroid while at P21, tT4 and tT3 levels in plasma were similar to C rats. Possible age and gender differences affecting thyroid hormone concentrations during development of the cerebral cortex of rats should be addressed in further studies. The adjustment of doses and their timing for T4 treatment of neonates diagnosed with congenital hypothyroid must be derived from the very important studies that correlate a child’s neurocognitive outcome with T4 treatments (Rovet, 2005), given that the levels of thyroid hormones in the cerebral cortex cannot be obtained.

### Association Between T_2_r and CC Maturation

There is a common consensus that water trapped in myelin and the reduction of the extra-cellular space are the main factors decreasing T_2_r values, which reflect the degree of maturation of the commissures in mammals (Le Bihan, 2014; Jelescu et al., 2016; MacKay and Laule, 2016). Supporting this, T_2_r was found related with the percentage and number of myelinated axons and axon density in the AC (Lucia et al., 2018), both of which contribute to the reduction of extra-cellular space. The tight association between T_2_r values and number of myelinated axons is clearly demonstrated in this work, indicating its usefulness in assessing myelination in children, by itself or in conjunction with myelin water fraction or magnetization transfer.

In the CC of C and MMI rats, T_2_r rapidly decreased from P8 to P20, and at a slower pace from P20 to P100 after which it leveled off, showing that at some point during cortical maturation, T_2_r becomes insensitive to white matter and extra-cellular changes. In fact, myelinated axon number continues to increase after P100 (more significantly in C rats, Figures 10B,D,F) in parallel with an increased number of glial cells (Gravel et al., 1990; Berbel et al., 1994). This is noteworthy since MRI techniques currently available may not be sensitive to changes in myelinated axon number in later periods of cortical maturation (especially to those of small diameter showing reduced myelin thickness), thereby limiting their early detection of white matter associated diseases. T_2_r images of children aged 6–48 months show a T_2_r sensitivity inflection point from 20 months onward, missing the myelination progression in subcortical white matter of the parietal and occipital lobe (Liu et al., 2018). In MRI scans, demyelination of callosal commissural and subcortical white matter axons in patients suffering toxic leucoencephalopathy only became detectable when the disease was at an advanced stage (Phan-Ba et al., 2012). In the case of deficient myelination, often seen using MRI in some young patients with monocarboxylate transporter 8 deficiency (Namba et al., 2008; Gika et al., 2010), current MRI techniques may not be able to follow the progression of demyelinating diseases, that become evident beyond 5–6 years of age (Vaurs-Barrière et al., 2009; Gika et al., 2010). As already discussed in the case of the anterior commissure (Lucia et al., 2018), this raises the important issue in MRI analysis of differentiation between delayed myelination and hypomyelination (van der Knaap and Wolf, 2010) in infants and children suffering diseases affecting white matter development. Enhanced approaches such as MRI through Mn^2+^ injection (MEMRI; Chan et al., 2012; Deng et al., 2019) and diffusion kurtosis image (DKI; Cheung et al., 2009) which is highly sensitive and directionally specific, should add valuable information to the study of developmental ultrastructural changes in white matter and the plasticity of callosal connections in normal and hypothyroid rats.

In MMI and C rats, the lowest T_2_r values are found in anterior CC and the highest in posterior CC, reflecting the antero-posterior gradient of maturation in the CC. Anterior (motor) and middle (somatosensory) callosal axons grow equally in diameter and myelinate earlier than posterior (auditory and visual) axons (Supplementary Figure 3 and Supplementary Table S5). In humans and monkeys, myelination begins in the body of the CC, then the splenium and continues in the genu (LaMantia and Rakic, 1990; Luders et al., 2010). In monkeys, this gradient parallels the growth in diameter of callosal axons, which in turn depends on the speed at which contralateral neocortical areas communicate (Caminiti et al., 2009; Innocenti, 2011). These data suggest a similar gradient in rats, humans and monkeys, given that the frontal telencephalic region in rats is principally a motor area (Krubitzer et al., 2011) with commissural neurons sending their axons through the anterior CC (Isseroff et al., 1984). Although the antero-posterior gradient is maintained in MMI rats, the maturation of the CC is delayed in MMI in comparison with C rats with the posterior zone being the most affected region (Supplementary Figure 2 and Supplementary Table S5). The maturation delay of the posterior CC can be explained by the severe alterations observed in visual and auditory neurons observed in hypothyroid rats (Ruiz-Marcos et al., 1979; Uziel et al., 1985). Our data strongly support the MRI data from children with early postnatal thyroid hormone insufficiency that show cognitive alterations involving language, motor, auditory and visual functions affecting telencephalic commissural connections (Rovet et al., 1992; Clairman et al., 2015).

### Abnormal Callosal Information Transfer in MMI Rats and Effects on Connectivity

Although an in-depth review on how development and evolution can drive the transfer of information within and between cerebral hemispheres in mammals is beyond the scope this study, we would point out that callosal connections are fundamental to the understanding of how emergent specialized cortical areas in more evolved mammals are connected (Kaas, 2013). The number and size of connected areas, the spread of information within these areas and the conduction delay to synchronize signals are all fundamental factors (Innocenti, 2017). These factors in turn depend on axon length and intermodal distances (Rushton, 1951; Waxman and Bennett, 1972; Waxman, 1975), size of terminal arbors and axon boutons, and the tangential and radial distribution of callosal neurons (Innocenti, 2011, 2017; Innocenti and Caminiti, 2017). Additional data suggests that the size (and conduction velocity) of myelinated callosal axons decreases as hemispheric specialization and asymmetry increases (Caminiti et al., 2009).

Significant differences in number and diameter of unmyelinated and myelinated axons were found between C and transient and chronic hypothyroid rats, which shows that the transfer of information between cerebral hemispheres in MMI rats is abnormal. Factors such as abnormal radial and tangential neuron location, terminal arbor size and bouton number of callosal axons are significantly affected in the cerebral cortex of transient and chronic hypothyroid rats (Berbel et al., 2014).

Although g-ratio was similar between MMI and C rats and to values obtained in other species (Waxman and Swadlow, 1976; Stikov et al., 2015), the distance between homotopic callosal areas decreased in MMI rats, with a corresponding decrease in conduction delays estimated from these distances and the outer diameter of myelinated axons. The size of terminal arbors and number of boutons in callosal axons of MMI rats might be also affected. The formation of microcircuits in the cerebral cortex is dependent on the activation of the sonic hedgehog signaling pathway (Harwell et al., 2012), which is regulated by T3 (Gil-Ibañez et al., 2017). In fact, the number and density of excitatory and inhibitory boutons in the somatosensory cortex of early postnatal hypothyroid rats (Navarro et al., 2015), and the number and length of terminal branches of thalamic axons, and the number of boutons in these branches were significantly reduced in chronic hypothyroid rats (Ausó et al., 2001) were observed.

Unmyelinated axon inner diameter in the anterior and posterior CC was less in chronic hypothyroid compared to transient hypothyroid and C rats (Supplementary Table S5). However, myelinated axon inner diameter in the anterior and middle CC was smaller in chronic hypothyroid compared to transient and C rats, while in the posterior CC, it was smaller in chronic and transient hypothyroid rats compared to controls. Furthermore, the estimated conduction velocity decreased more in the posterior CC of MMI rats than in middle and anterior CC, compared to C rats. These data strongly suggest a delay in CC transfer of information in chronic and transient hypothyroid rats, being more pronounced in the posterior CC.

The estimated conduction delay of myelinated axons between homotopic callosal neurons based on morphological characteristics is speculative because it is not based on electrophysiological recordings. Conduction velocity is estimated on precise EM data from myelinated axons, but other data such as: (i) type of myelinated axon (e.g., motor, somatosensorial, visual, and auditory callosal axons) and (ii) the individual length of myelinated axons, could not be fully ascertained.

Electrophysiological recordings showing properties of callosal axons from living cells are unfortunately not available in early postnatal transient hypothyroid rats. Our estimations are based on the methodology published in pioneering work (Rushton, 1951; Waxman and Bennett, 1972; Waxman, 1975) and more recent electrophysiological and anatomical combined studies (Roxin et al., 2005; Caminiti et al., 2009, 2013). We provide data that may help understand the role of thyroid hormones in the transfer of information between cortical hemispheres of rats at earlier postnatal ages.

The conduction delay of a myelinated axon reflects the time needed for an action potential to reach its target. Conduction delay is calculated as axon length divided by conduction velocity (= 5.5 × myelinated axon outer diameter). Velocity and length have opposite effects: if velocity (axon diameter) is greater then delay is less, conversely if length is greater then delay is greater. Our data suggests that in MMI rats the distances between hemispheres are more affected (they have smaller brains) than the myelinated axon conduction velocity. On average, the distance between cortical areas decreased 12.1 ± 5.0% in transient and 22.1 ± 6.6% in chronic hypothyroid rats, while the conduction velocity decreased 4.7 ± 2.5% in transient and 11.5 ± 2.9% in chronic hypothyroid rats with respect to controls (Supplementary Table S6).

### Altered Behavior

The decreased number of myelinated axons in the CC of MMI rats will affect motor, somatosensorial, auditory and visual inputs, jeopardizing the spatial and social learning of MMI rats and consequently, its behavior.

As discussed in our previous study (Navarro et al., 2015), specific tests have been undertaken to determine the effects of cerebral cortex alterations in hypothyroid rats. These tests studied audiogenic seizure susceptibility and motor excitability (Van Middlesworth and Norris, 1980; Ng et al., 2001; Ausó et al., 2004; Wallis et al., 2008). Hearing loss is a common neurologic disease found in humans with different types of hypothyroidism such as cretinism (Trotter, 1960; DeLong et al., 1985; Morreale de Escobar et al., 2000), congenital hypothyroidism (Vanderschueren-Lodeweyckx et al., 1983; Rovet et al., 1996) and resistance to thyroid hormone also known as Refetoff syndrome (Refetoff et al., 1967). Deafness caused by hypothyroidism is often due to irreversible damage of the Corti’s organ (Knipper et al., 2000) and to abnormal development of the brain stem (Halpern et al., 1989; Ma et al., 1989). Impaired hearing (Navarro et al., 2015) and the abnormal radial distribution of callosal auditory neurons (Berbel et al., 1993) have been found in chronic hypothyroid rats. The delay observed in the maturation of the posterior CC in transient and chronic hypothyroid rats in the present work is an additional factor to be considered for understanding the physiopathology of hearing loss in late treated children with congenital hypothyroidism.

The study of learning, attention and memory deficits revealed alterations in the organization of the cerebral cortex, including callosal connections (Negishi et al., 2005; Venero et al., 2005; Gilbert et al., 2007; Opazo et al., 2008; Gilbert and Lasley, 2013). In mammals, prepulse inhibition of the acoustic startle response measures sensorimotor gating mechanisms (Graham, 1975; Blumenthal et al., 1996; Koch, 1999). Deficient sensorimotor gating, demonstrated by disrupted prepulse inhibition, exists in several neuropsychiatric disorders such as schizophrenia (Braff et al., 2001; Hamm et al., 2001). In hypothyroid rats from P0 to P40, reduced prepulse inhibition of the acoustic startle response was found to be stimulus intensity dependent (Navarro et al., 2015), and is associated with decreased electrophysiological activity of the somatosensory cortex (Graham, 1975; Blumenthal et al., 1996; Koch, 1999), which might reflect a degree of attention deficit, including impaired spatial learning (Wilcoxon et al., 2007). This is in agreement with elevated open arms test data (Navarro et al., 2015) where MMI pups typically walked to the end of the open arm and then fell, suggesting an orientation and memory loss concerning dangerous situations in which the somatosensory cortex could be involved. This possibility is supported by data showing abnormal cytoarchitecture and connectivity in the primary somatosensory cortex of MMI rats (Berbel et al., 2001; Ausó et al., 2001) and the impaired callosal connections identified in this paper.

### Implications for Neurological and Psychiatric Diseases in Humans

The use of animal models to further our understanding of the physiopathology of neurocognitive and psychiatric diseases has its limitations. Most of these diseases are caused by alterations of cortical areas not found in lower vertebrates (e.g., the frontal and prefrontal cortices). The frontal and prefrontal areas of the rat are greatly reduced (as further discussed below) and consequently, the genu contains mostly motor and some somatosensory axons, instead of the prefrontal and frontal callosal axons that are found in the human’s genu. The abnormal development of the neocortex is associated with childhood and adolescent neurocognitive (Goodman et al., 2014; Wheeler et al., 2015) and psychiatric disorders such as autism spectrum disorders (ASD; Bauman and Kemper, 2005; Román et al., 2013), attention deficit-hyperactivity disorder (ADHD; Li Y. et al., 2014; Li Z. et al., 2014) and schizophrenia (Innocenti et al., 2003; Santos et al., 2012). In particular, alterations in cerebral white matter have been described in ASD (Piven et al., 1997; Alexander et al., 2007), ADHD (Hynd et al., 1991), bipolar disorder and schizophrenia (McIntosh et al., 2009; Santos et al., 2012). In all these disorders, events such as abnormal cell proliferation and migration, deficient axonal branching and growth, myelination and oligodendrocyte development, unbalanced excitatory/inhibitory input ratios and abnormal firing synchronization may occur.

Although there are important differences in the development of the cerebral cortex between humans and rats, similarities can be found in the basic events of corticogenesis such as axonal sprouting and pruning, myelination and synaptogenesis, as well as cortical functions that are driven by evolutionary preserved T3-regulated genes (Morte et al., 2010; Berbel et al., 2014; Chatonnet et al., 2015; Bernal, 2017). To this extent we can consider that the transiently hypothyroid rats in this study mimic the condition of late diagnosed congenital hypothyroidism in children, resulting in impaired cognitive development (O’Callaghan et al., 1995; Kester et al., 2004; Rovet and Simic, 2008; Williams and Hume, 2008; Willoughby et al., 2014).

In cultures of cortical cells derived from E14 mice several hundred genes transcriptionally regulated by T3 have been described as being involved in key events of cerebral cortex development (Morte et al., 2010; Berbel et al., 2014; Chatonnet et al., 2015; Gil-Ibañez et al., 2017; Bernal, 2017). Some of these genes have been found mutated in humans diagnosed with neurological and mental diseases (Berbel et al., 2014; Gil-Ibañez et al., 2017; Bernal, 2017). Cortical white matter abnormalities have been described in bipolar disorders and schizophrenia, and have been associated with an abnormal expression of neuregulin-1, which plays a crucial role in oligodendrocyte development and function (Fernandez et al., 2000; McIntosh et al., 2009; Uranova et al., 2018). Genes involved in the expression of neuron-glia adhesion proteins affecting myelination are under-expressed in schizophrenia and bipolar disorder (Tkachev et al., 2003) as well as in postnatal hypothyroid rats (Rodríguez-Peña et al., 1993; Rodríguez-Peña, 1999; Barradas et al., 2001; Sharlin et al., 2008). Furthermore, Erk1/2 expression is decreased in late fetal hypothyroid rats (Berbel et al., 2010). The Erk1/2 pathway activates autotaxin, which drives oligodendrocyte maturation (Liu et al., 2011; Lee and Petratos, 2016). All these data suggest that the decreased number and percentage of myelinated axons in transient and chronic hypothyroid rats might result not only from arrested axon growth, resulting in small diameter callosal axons, but also that congenital hypothyroidism might also affect the proliferation and maturation of oligodendrocyte precursors, albeit transiently. This would explain at least in part, the physiopathology of myelinated tracts in the cerebral cortex of late diagnosed children with congenital hypothyroidism, and the relevance of genes involved in the myelination of cortical white matter axons. Any factor altering the expression of T3-regulated genes associated with the development of telencephalic commissures, such as low T3 availability to target cells, must be considered as potentially increasing the risk of children developing neurologic and psychiatric diseases.

## Conclusion

Early postnatal hypothyroidism decreases axon number and percentage in the CC, preventing the decay of T_2_r values. The regression functions obtained from T_2_r values in C rats are a good tool for estimating the number and percentage of myelinated axons at earlier postnatal ages.

EM data shows that the total number of axons in the CC at P150 is similar in MMI and C rats, while myelinated axon number and percentage is reduced in MMI rats. Conduction velocity is reduced in MMI rats. These data reflect an alteration in the flow of information between cortical areas connected through the CC, which may in turn affect neurocognitive function. The quantitative EM data reported here shows the importance of thyroid hormones at early postnatal ages for a normal CC development, and supports the findings of MRI data related to cognitive alteration in children with early postnatal thyroid hormone insufficiency.

Despite the clear limitations imposed by animal models, our results will nonetheless help to better understand the role of thyroid hormones in impaired cognitive development, found in new born children suffering from congenital hypothyroidism, children breastfed by mothers suffering postpartum thyroiditis, or iodine deficiency (mother and neonate), a condition which is often late diagnosed.

## Data Availability Statement

All datasets generated for this study are included in the article/Supplementary Material.

## Ethics Statement

The animal study was reviewed and approved by the Ethics Committee of the University Miguel Hernández of Elche, Alicante and the Generalitat of València, València, Spain.

## Author Contributions

PB carried out the conception, design, and draft of the manuscript. FS-L, JP-T, and PB contributed to the acquisition, analysis, and interpretation of MRI data. FS-L, SG-G, JG-V, and PB contributed to the acquisition, analysis, and interpretation of EM data. All authors contributed to the discussion of the results, writing specific parts of the manuscript, and final manuscript approval.

## Conflict of Interest

The authors declare that the research was conducted in the absence of any commercial or financial relationships that could be construed as a potential conflict of interest.
