# Supplementary material for: Transient Hypothyroidism During Lactation Alters the Development of the Corpus Callosum in Rats. An in vivo Magnetic Resonance Image and Electron Microscopy Study
Source: Front Neuroanat. 2020 Jun 26;14:33. doi: 10.3389/fnana.2020.00033 (PMC7333461; doi:10.3389/fnana.2020.00033)
Supplement: Supplementary file 1 [file Data_Sheet_1.PDF]

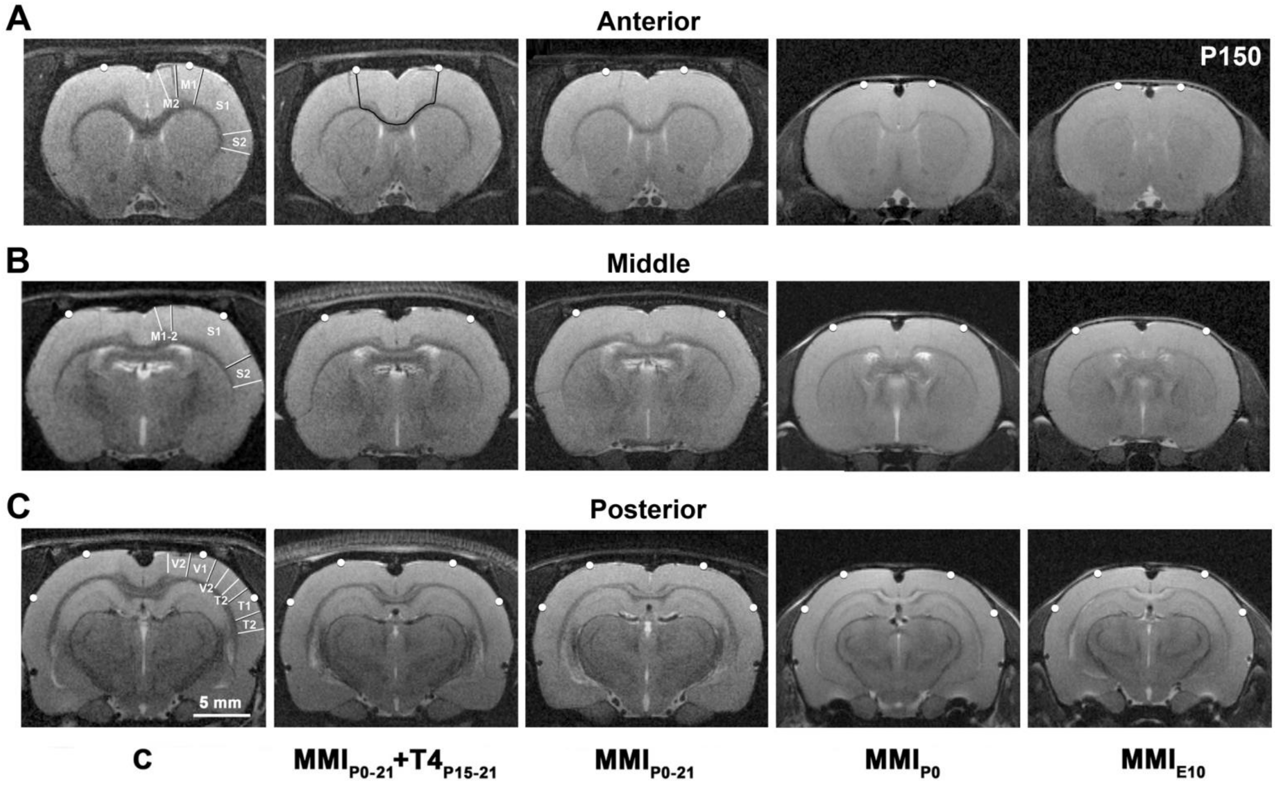

Supplementary Figure S1. Coronal T2w images of MMI and C rats at P150 from anterior **(A)**, middle **(B)** and posterior **(C)** CC. The T2w data were obtained from P150C rats at the distances from Bregma shown in **Figure 2 B-D**. At these distances, motor (M1-2), somatosensory (S1-S2), visual (V1-V2) and auditory (T1-T2) cortices (delimited by white lines) are shown in the control sections (Paxinos et al., 2015). Dots at the pial surface mark the midpoint of motor (M1-2), primary somatosensory (S1), visual (V1) and auditory (T1) areas respectively. Due to brain shrinkage in MMI rats, their coronal sections were compared to control sections using anatomical landmarks. **(A)** The anterior landmark in C rats is at 0.89 mm from Bregma, where the optic nerves begin to form the optic chiasm; the middle landmark in C rats is at -1.61 mm from Bregma, where the rostral hippocampus begins to appear; and the posterior landmark in C rats is at -4.8 mm from Bregma where the caudal end of the hippocampal commissure begins to appear. The distances between like cortical areas were measured from homotopic dots (an example traced by a black line is shown in the anterior section of the MMI_{P0-P21}+$T4_{P15-P21}$ rat). Note the reduced size of MMI rats compared to controls. Same scale for all images.
