# Supplementary material for: Transient Hypothyroidism During Lactation Alters the Development of the Corpus Callosum in Rats. An in vivo Magnetic Resonance Image and Electron Microscopy Study
Source: Front Neuroanat. 2020 Jun 26;14:33. doi: 10.3389/fnana.2020.00033 (PMC7333461; doi:10.3389/fnana.2020.00033)
Supplement: Supplementary file 2 [file Data_Sheet_2.PDF]

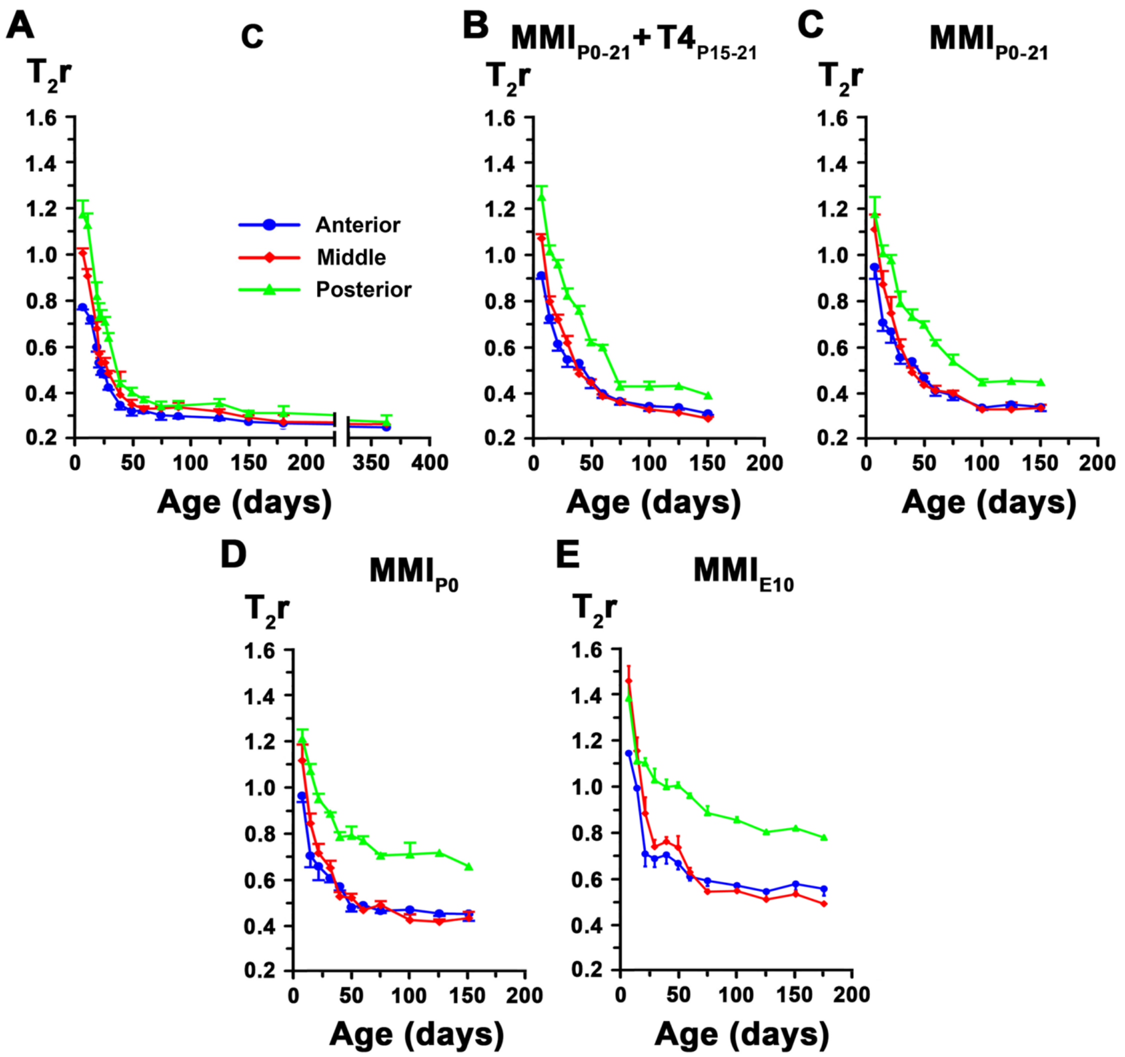

Supplementary figure S2. T_2r of the anterior, middle and posterior CC at various postnatal ages. In C rats (**A**), anterior CC T_2r values are the lowest, and posterior values the highest. Transient (**B,C**), and chronic (**D,E**) hypothyroid rats show a similar trend but with T_2r values significantly higher than control. These differences were more exagerated in the posterior CC of MMI rats.
