# Supplementary material for: Transient Hypothyroidism During Lactation Alters the Development of the Corpus Callosum in Rats. An in vivo Magnetic Resonance Image and Electron Microscopy Study
Source: Front Neuroanat. 2020 Jun 26;14:33. doi: 10.3389/fnana.2020.00033 (PMC7333461; doi:10.3389/fnana.2020.00033)
Supplement: Supplementary file 3 [file Data_Sheet_3.PDF]

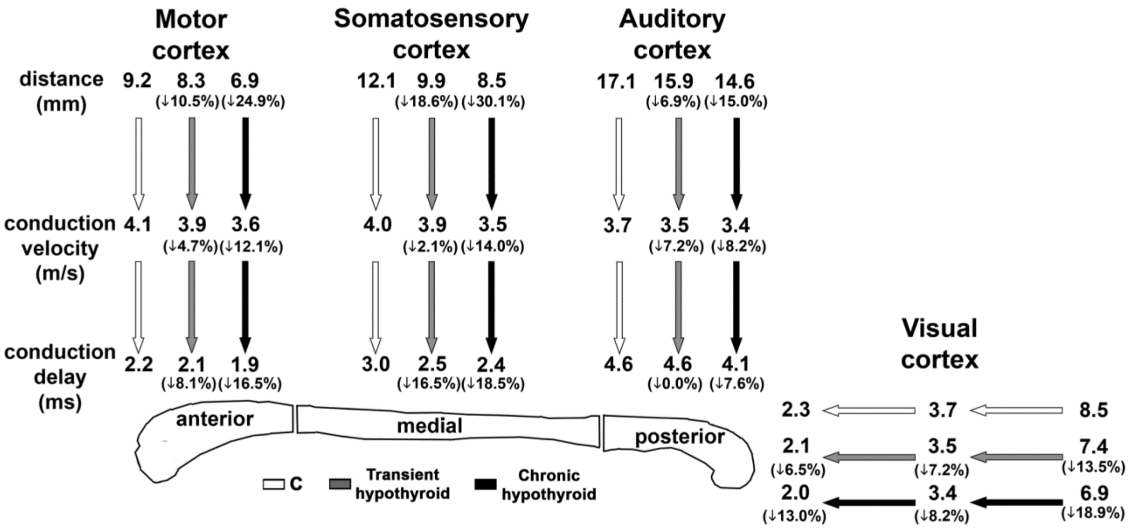

Supplementary Figure S3. The figure shows the estimated length (distance), conduction velocity (cv) and conduction delay (cd) of myelinated callosal axons from homotopic primary motor and sensory cortical areas, that pass through anterior, medial and posterior zones (shown in the CC outline) for C (white arrows), transient (grey arrows) and chronic hypothyroid (black arrows) rats at P150. Distance is an estimation of the mean length of callosal axons assuming that their parent callosal neurons and targets are situated in cortical layer I near the pia, of each cortical area. Conduction velocity depends on myelinated axon outer diameter and g-ratio (inner to outer diameter ratio). Conduction delay indicates the time calculated for action potentials to travel the estimated length of myelinated axons and is obtained from the ratio of distance to conduction velocity (conduction delay = distance / conduction velocity). Consequently, the conduction delay is dependent on variations in the length of callosal axons (greater axon length increases the time taken for the signal to reach its target, assuming a constant conduction velocity), and changes in the outer diameter of axons (increased outer diameter and g-ratio confers greater conduction velocity and a reduction in conduction delay, assuming distance remains constant).

The percentage values shown in brackets indicate the average relative decrease of these parameters in transient and chronic hypothyroid rats with respect to controls. In MMI rats, both conduction velocity and conduction delay decreased in all CC zones (except for auditory callosal axons in transient hypothyroid rats). This being possible due to the significant brain shrinkage and consequent reduction in axon length observed in MMI rats (with the greatest effect observed in chronic hypothyroid rats). Chronic hypothyroid rats had a similar conduction velocity in all zones of the CC (ranging from 3.6 m/s in anterior to 3.4 m/s in medial and posterior zones) and values were lower than those of transient hypothyroid and control rats. Transient hypothyroid rat velocity was the same in anterior and medial zones (3.9 m/s), and reduced in posterior zones (3.5 m/s), indicating an improved myelination of callosal axons, not sufficient however, to compensate the effects of brain shrinkage on conduction delay.
