# Supplementary material for: Transient Hypothyroidism During Lactation Alters the Development of the Corpus Callosum in Rats. An in vivo Magnetic Resonance Image and Electron Microscopy Study
Source: Front Neuroanat. 2020 Jun 26;14:33. doi: 10.3389/fnana.2020.00033 (PMC7333461; doi:10.3389/fnana.2020.00033)
Supplement: Supplementary file 4 [file Data_Sheet_4.PDF]

Supplementary Table S1: T_2r values in the anterior CC at different postnatal ages.

Age (days)	C	MMI_{P0-21} $+T4_{P15-21}$	MMI_{P0-21}	MMI_{P0}	MMI_{E10}
P8	0.77 ± 0.03[a]	0.91 ± 0.01[a]	0.95 ± 0.05[a]	0.97± 0.03[a]	1.15 ± 0.05[a]
P15	0.72 ± 0.04[a]	0.73 ± 0.02[a]	0.71 ± 0.04[a]	0.73 ± 0.05[a]	1.00 ± 0.05[a]
P20	0.60 ± 0.02[b]				
P22	0.53 ± 0.01[b]	0.62 ± 0.03[b]	0.67 ± 0.05[a]	0.66 ± 0.06[a]	0.71 ± 0.04[a]
P24	0.49 ± 0.02[b]				
P27	0.48 ± 0.02[b]				
P30	**0.42 ± 0.02[c]**	0.55 ± 0.03[b]	0.56 ± 0.03[b]	0.61 ± 0.02[a]	0.69 ± 0.02[a]
P40	**0.35 ± 0.01[c]**	0.53 ± 0.02[b]	0.54 ± 0.02[b]	0.57 ± 0.02[b]	0.71 ± 0.03[a]
P50	**0.32 ± 0.01[c]**	**0.45 ± 0.03[c]**	0.47 ± 0.02[b]	0.48 ± 0.02[b]	0.67 ± 0.02[a]
P60	**0.32 ± 0.01[c]**	**0.40 ± 0.02[c]**	**0.41 ± 0.02[c]**	0.49 ± 0.01[b]	0.61 ± 0.02[b]
P75	**0.30 ± 0.01[c]**	**0.37 ± 0.02[c]**	**0.39 ± 0.02[c]**	0.46 ± 0.01[b]	0.60 ± 0.05[b]
P100	**0.30 ± 0.02[c]**	**0.34 ± 0.02[c]**	**0.34 ± 0.01[c]**	**0.47 ± 0.02[c]**	0.57 ± 0.04[b]
P125	**0.29 ± 0.01[c]**	**0.34 ± 0.01[c]**	**0.35 ± 0.01[c]**	**0.45 ± 0.01[c]**	0.55 ± 0.04[b]
P150	**0.27 ± 0.02[c]**	**0.31 ± 0.01[c]**	**0.34 ± 0.02[c]**	**0.43 ± 0.03[c]**	0.58 ± 0.02[b]
P180	**0.26 ± 0.01[c]**				0.56 ± 0.02[b]
P365	**0.25 ± 0.01[c]**				

Values are mean ± SD (n = 8). The anterior CC is lightly ([a]), similar ([b]) and darker ([c]) than the adjacent neuropil.
