# Supplementary material for: Transient Hypothyroidism During Lactation Alters the Development of the Corpus Callosum in Rats. An in vivo Magnetic Resonance Image and Electron Microscopy Study
Source: Front Neuroanat. 2020 Jun 26;14:33. doi: 10.3389/fnana.2020.00033 (PMC7333461; doi:10.3389/fnana.2020.00033)
Supplement: Supplementary file 5 [file Data_Sheet_5.PDF]

**Supplementary Table S2:** $T_2$r values in the middle CC at different postnatal ages.

| Age (days) | C | MMI$_{P0-21}$ +T4$_{P15-21}$ | MMI$_{P0-21}$ | MMI$_{P0}$ | MMI$_{E10}$ |
|---|---|---|---|---|---|
| P8 | 1.01 ± 0.02[a] | 1.08 ± 0.02[a] | 1.11 ± 0.06[a] | 1.12 ± 0.07[a] | 1.46 ± 0.05[a] |
| P15 | 0.91 ± 0.03[a] | 0.80 ± 0.02[a] | 0.87 ± 0.06[a] | 0.85± 0.04[a] | 1.16 ± 0.04[a] |
| P20 | 0.68 ± 0.03[a] | | | | |
| P22 | 0.57 ± 0.01[b] | 0.72 ± 0.02[a] | 0.75 ± 0.05[a] | 0.72 ± 0.04[a] | 0.89 ± 0.04[a] |
| P24 | 0.53 ± 0.02[b] | | | | |
| P27 | 0.53 ± 0.02[b] | | | | |
| P30 | 0.48 ± 0.01[b] | 0.62 ± 0.03[b] | 0.61 ± 0.02[b] | 0.65 ± 0.03[b] | 0.74 ± 0.03[a] |
| P40 | **0.39 ± 0.01[c]** | 0.49 ± 0.02[b] | 0.49 ± 0.01[b] | 0.53 ± 0.02[b] | 0.77 ± 0.02[a] |
| P50 | **0.35 ± 0.02[c]** | **0.45 ± 0.01[c]** | **0.44 ± 0.02[c]** | 0.52 ± 0.02[b] | 0.74 ± 0.02[a] |
| P60 | **0.33 ± 0.01[c]** | **0.39 ± 0.01[c]** | **0.41 ± 0.01[c]** | 0.47 ± 0.02[b] | 0.63 ± 0.02[b] |
| P75 | **0.33 ± 0.01[c]** | **0.36 ± 0.01[c]** | **0.40 ± 0.04[c]** | 0.49 ± 0.02[b] | 0.55 ± 0.04[b] |
| P100 | **0.33 ± 0.02[c]** | **0.33 ± 0.02[c]** | **0.33 ± 0.01[c]** | **0.42 ± 0.03[c]** | 0.55 ± 0.03[b] |
| P125 | **0.32 ± 0.01[c]** | **0.32 ± 0.02[c]** | **0.33 ± 0.02[c]** | **0.42 ± 0.01[c]** | 0.51 ± 0.03[b] |
| P150 | **0.29 ± 0.01[c]** | **0.31 ± 0.01[c]** | **0.34 ± 0.02[c]** | **0.43 ± 0.03[c]** | 0.54 ± 0.05[b] |
| P180 | **0.28 ± 0.03[c]** | | | | 0.50 ± 0.02[b] |
| P365 | **0.26 ± 0.01[c]** | | | | |

Values are mean ± SD (n = 8). The anterior CC is lightly ([a]), similar ([b]) and darker ([c]) than the adjacent neuropil.
