# Supplementary material for: Transient Hypothyroidism During Lactation Alters the Development of the Corpus Callosum in Rats. An in vivo Magnetic Resonance Image and Electron Microscopy Study
Source: Front Neuroanat. 2020 Jun 26;14:33. doi: 10.3389/fnana.2020.00033 (PMC7333461; doi:10.3389/fnana.2020.00033)
Supplement: Supplementary file 7 [file Data_Sheet_7.PDF]

Supplementary Table S4. CC Mid-sagittal area, axon number and percentage at P150.

	C	MMI_{P0-21} + $T4_{P15-21}$	MMI_{P0-21}	MMI_{P0}	MMI_{E10}
**Anterior**					
Area (µm^2) (x10^3)	831.8 ± 120.4	604.5 ± 51.5	691.6 ± 132.7	309.8 ± 34.6	330.8 ± 39.6
Total ax. no. (x10^3)	3712.9 ± 716.8	3798.6 ± 349.9	3647.2 ± 568.8	3303.8 ± 234.2	4029.5 ± 482.0
Unmyel. ax. no. (x10^3)	2651.3 ± 638.6	2976.8 ± 301.8	2874.7 ± 654.1	3070.3 ± 69.8	3892.0 ± 48.5
Myel. ax. no. (x10^3)	1061.6 ± 73.4	821.8 ± 94.7	772.5 ± 124.3	233.5 ± 58.1	140.2 ± 40.8
Myel. ax. %	31.8 ± 6.6	22.6 ± 3.4	21.8 ± 7.6	7.2 ± 1.1	3.7 ± 0.1
Est. myel. ax. no. (x10^3)	1097.8 ± 81.3	873.8 ± 20.4	734.6 ± 43.1	378.9 ± 26.4	156.3 ± 6.5
**Middle**					
Area (µm^2) (x10^3)	985.0 ± 218.4	826.7 ± 53.5	782.2 ± 70.6	344.2 ± 47.1	369.7 ± 50.8
Total ax. no. (x10^3)	4931.0 ± 465.2	5164.5 ± 349.9	4747.8 ± 668.2	4329.2 ± 406.9	5213.4 ± 179.9
Unmyel. ax. no. (x10^3)	3485.5 ± 703.8	3867.6 ± 862.4	3693.8 ± 616.6	4028.9 ± 410.0	4977.5 ± 164.1
Myel. ax. no. (x10^3)	1445.5 ± 188.7	1296.9 ± 161.4	1054.0 ± 168.6	300.3 ± 65.2	235.9 ± 28.6
Myel. ax. %	32.3 ± 6.9	25.4 ± 6.3	21.1 ± 3.0	7.6 ± 1.1	4.8 ± 0.5
Est. myel. Ax. no. (x10^3)	1436.0 ± 49.5	1244.7 ± 40.2	1023.6 ± 60.7	528.1 ± 36.8	245.5 ± 9.1
**Posterior**					
Area (µm^2) (x10^3)	722.7 ± 145.4	474.1 ± 89.7	487.8 ± 33.8	267.8 ± 33.2	291.9 ± 38.1
Total ax. no. (x10^3)	4718.5 ± 568.8	5895.0 ± 285.1	5601.6 ± 869.1	5073.2 ± 848.7	5670.0 ± 24.3
Unmyel. ax. no. (x10^3)	3572.4 ± 843.7	5353.1 ± 289.6	5122.7 ± 532.2	5002.8 ± 843.2	5625.0 ± 79.5
Myel. ax. no. (x10^3)	1146.1 ± 174.7	541.9 ± 41.3	478.9 ± 32.2	70.4 ± 5.4	45.0 ± 10.6
Myel. ax. %	28.1 ± 4.0	9.5 ± 0.5	8.7 ± 2.0	1.7 ± 0.3	0.9 ± 0.2
Est. myel. ax. no. (x10^3)	1123.5 ± 37.4	551.9 ± 14.1	384.2 ± 17.1	114.2 ± 4.8	77.0 ± 1.9
**CC (all zones)**					
Area (µm^2) (x10^3)	2539.5 ± 479.7	1905.3 ± 181.0	1961.6 ± 210.9	921.8 ± 90.5	992.5 ± 119.1
Total ax. no. (x10^3)	13362.2 ± 888.9	14858.2 ± 283.2	13996.5 ± 868.3	12706.2 ± 426.3	14913.1 ± 841.5
Unmyel. ax. no. (x10^3)	9709.1± 854.5	12197.6 ± 297.8	11691.1 ± 920.9	12102.0 ± 441.1	14494.6 ± 97.4
Myel. ax. no. (x10^3)	3653.1 ± 145.6	2660.6 ± 76.9	2305.4 ± 108.4	604.2 ± 22.3	418.5 ± 26.7
Myel. ax. %	27.3 ± 5.8	17.9 ± 2.4	16.5 ± 4.2	4.8 ± 0.8	2.8 ± 0.1

Data are mean ± SD. Four rats per group. Ax.: axon. Unmyel.: unmyelinated. Myel.: myelinated. Est.: estimated.
