# Supplementary material for: Transient Hypothyroidism During Lactation Alters the Development of the Corpus Callosum in Rats. An in vivo Magnetic Resonance Image and Electron Microscopy Study
Source: Front Neuroanat. 2020 Jun 26;14:33. doi: 10.3389/fnana.2020.00033 (PMC7333461; doi:10.3389/fnana.2020.00033)
Supplement: Supplementary file 8 [file Data_Sheet_8.pdf]

Supplementary Table S5. Axon diameter (μm), myelin thickness (μm), g-ratio and conduction velocity (m/s) in the CC at P150.

	C	MMI_{P0-21} + $T4_{P15-21}$	MMI_{P0-21}	MMI_{P0}	MMI_{E10}
**Anterior**					
Unmyel. axon diam. (median)	0.24 ± 0.07 (0.22)	0.24 ± 0.08 (0.22)	0.25 ± 0.07 (0.24)	0.21 ± 0.07 (0.21)	0.16 ± 0.04 (0.16)
Myel. axon inner diam. (median)	0.58 ± 0.20 (0.54)	0.55 ± 0.20 (0.52)	0.54 ± 0.19 (0.53)	0.49 ± 0.13 (0.48)	0.49 ± 0.15 (0.47)
Myelin thickness (median)	0.084 ± 0.026 (0.078)	0.081 ± 0.023 (0.078)	0.080 ± 0.020 (0.076)	0.081 ± 0.019 (0.077)	0.084 ± 0.021 (0.079)
g-ratio (median)	0.77 ± 0.06 (0.77)	0.76 ± 0.07 (0.77)	0.76 ± 0.06 (0.77)	0.75 ± 0.06 (0.75)	0.74 ± 0.07 (0.75)
Velocity (median)	4.09 ± 1.30 (3.86)	3.92 ± 1.22 (3.76)	3.87 ± 1.12 (3.72)	3.57 ± 0.77 (3.48)	3.62 ± 0.89 (3.54)
**Middle**					
Unmyel. axon diam. (median)	0.20 ± 0.08 (0.18)	0.20 ± 0.07 (0.18)	0.20 ± 0.07 (0.19)	0.19 ± 0.06 (0.18)	0.18 ± 0.05 (0.17)
Myel. axon inner diam. (median)	0.56 ± 0.21 (0.52)	0.56 ± 0.22 (0.53)	0.56 ± 0.22 (0.51)	0.48 ± 0.14 (0.46)	0.46 ± 0.13 (0.45)
Myelin thickness (median)	0.085 ± 0.027 (0.079)	0.077 ± 0.023 (0.072)	0.077 ± 0.020 (0.074)	0.082 ± 0.023 (0.078)	0.080 ± 0.019 (0.078)
g-ratio (median)	0.76 ± 0.06 (0.76)	0.77 ± 0.06 (0.78)	0.77 ± 0.06 (0.78)	0.74 ± 0.06 (0.74)	0.73 ± 0.06 (0.74)
Velocity (median)	4.02 ± 1.34 (3.79)	3.94 ± 1.37 (3.74)	3.93 ± 1.34 (3.59)	3.53 ± 0.87 (3.40)	3.38 ± 0.78 (3.32)
**Posterior**					
Unmyel. axon diam. (median)	0.18 ± 0.06 (0.18)	0.17 ± 0.06 (0.16)	0.18 ± 0.06 (0.17)	0.17 ± 0.05 (0.16)	0.16 ± 0.04 (0.16)
Myel. axon inner diam. (median)	0.50 ± 0.16 (0.48)	0.48 ± 0.15 (0.46)	0.47 ± 0.15 (0.46)	0.45 ± 0.12 (0.43)	0.48 ± 0.12 (0.46)
Myelin thickness (median)	0.084 ± 0.025 (0.079)	0.078 ± 0.021 (0.075)	0.073 ± 0.017 (0.071)	0.087 ± 0.024 (0.082)	0.084 ± 0.021 (0.079)
g-ratio (median)	0.74 ± 0.06 (0.75)	0.75 ± 0.06 (0.75)	0.75 ± 0.06 (0.76)	0.73 ± 0.06 (0.72)	0.74 ± 0.06 (0.74)
Velocity (median)	3.70 ± 1.05 (3.53)	3.49 ± 0.89 (3.40)	3.38 ± 0.89 (3.27)	3.41 ± 0.79 (3.31)	3.58 ± 0.74 (3.41)

Data are mean ± SD. Four rats per group. Diam.: diameter. Myel.: myelinated. Unmyel.: unmyelinated.
