# Supplementary material for: Transient Hypothyroidism During Lactation Alters the Development of the Corpus Callosum in Rats. An in vivo Magnetic Resonance Image and Electron Microscopy Study
Source: Front Neuroanat. 2020 Jun 26;14:33. doi: 10.3389/fnana.2020.00033 (PMC7333461; doi:10.3389/fnana.2020.00033)
Supplement: Supplementary file 9 [file Data_Sheet_9.PDF]

Supplementary Table S6. Distance (mm), velocity (m/s) and conduction delay (ms) between areas projecting through the anterior, middle and posterior CC in C and MMI rats at P150.

	C	MMI_{P0-21} + $T4_{P15-21}$	MMI_{P0-21}	MMI_{P0}	MMI_{E10}
**Anterior**					
**Distance (M1)**	9.2 ± 0.4	8.3 ± 0.4	8.2 ± 0.3	7.0 ± 0.4	6.8 ± 0.1
Decrease		**10.3%**	**10.6%**	**23.6%**	**26.1%**
**Velocity**	4.1 ± 1.3	3.9 ± 1.2	3.9 ± 1.1	3.6 ± 0.8	3.6 ± 0.9
Decrease		**4.1%**	**5.3%**	**12.7%**	**11.4%**
**Conduction delay**	2.3 ± 0.1	2.1 ± 0.1	2.1 ± 0.1	2.0 ± 0.1	1.9 ± 0.1
Decrease		**8.5%**	**7.6%**	**14.5%**	**18.4%**
**Middle**					
**Distance (S1)**	12.1 ± 0.2	9.9 ± 0.5	9.8 ± 0.5	8.9 ± 0.2	8.1 ± 0.4
Decrease		**18.4%**	**18.8%**	**26.9%**	**33.3%**
**Velocity**	4.0 ± 1.3	3.9 ± 1.4	3.9 ± 1.3	3.5 ± 0.9	3.4 ± 0.8
Decrease		**2.1%**	**2.1%**	**12.1%**	**15.8%**
**Conduction delay**	3.0 ± 1.3	2.5 ± 0.1	2.5 ± 0.1	2.5 ± 0.1	2.4 ± 0.1
Decrease		**16.4%**	**16.8%**	**16.5%**	**20.6%**
**Posterior**					
**Distance (T1)**	17.1 ± 0.5	16.2 ± 0.3	15.7 ± 0.3	14.8 ± 0.8	14.3 ± 0.3
Decrease		**5.6%**	**8.2%**	**13.6%**	**16.4%**
**Velocity**	3.7 ± 1.1	3.5 ± 0.9	3.4 ± 0.9	3.4 ± 0.8	3.4 ± 0.7
Decrease		**5.7%**	**8.6%**	**7.8%**	**8.6%**
**Conduction delay**	4.6 ± 0.1	4.6 ± 0.1	4.7 ± 0.1	4.3 ± 0.2	4.2 ± 0.1
Decrease		**0.0%**	**0.0%**	**6.5%**	**8.7%**
**Distance (V1)**	8.5 ± 0.4	7.4 ± 0.3	7.4 ± 0.2	7.2 ± 0.7	6.6 ± 0.3
Decrease		**13.5%**	**13.5%**	**15.8%**	**22.0%**
**Velocity**	3.7 ± 1.1	3.5 ± 0.9	3.4 ± 0.9	3.4 ± 0.8	3.4 ± 0.7
Decrease		**5.7%**	**8.6%**	**7.8%**	**8.6%**
**Conduction delay**	2.3 ± 0.1	2.1 ± 0.1	2.2 ± 0.1	2.1 ± 0.2	1.9 ± 0.1
Decrease		**8.7%**	**4.3%**	**8.7%**	**17.3%**

The relative decrease of MMI rats with respect to controls (%) is indicated. M1, motor cortex. S1, somatosensorial cortex. T1, auditory cortex. V1, visual cortex.
